# In situ Surface Polishing Enables *V_OC_* Approaching 95% of the Theoretical Limit for Efficient Inverted Perovskite Solar Cells

**DOI:** 10.1002/advs.202503342

**Published:** 2025-03-31

**Authors:** Han‐Wen Zhang, Yan‐Gang Bi, Yi‐Fan Wang, Mu Lin, Chang Liu, Jing Feng

**Affiliations:** ^1^ State Key Laboratory of Integrated Optoelectronics College of Electronic Science and Engineering Jilin University 2699 Qianjin Street Changchun 130012 China

**Keywords:** 1, 1, 1, 3, 3, 3‐hexafluoropropan‐2‐ol, additive‐free, in situ surface polishing, perovskite solar cells, surface reconstruction

## Abstract

Surface modification of perovskite films by conventional chemical additives suffers from the complexity of the process and the risk of secondary contamination, which limits the open‐circuit voltage (*V_OC_*) of the perovskite solar cells (PSCs). The utilization of the clean‐passivate strategy for the polishing of perovskite films has garnered considerable attention in recent years. Nevertheless, this method necessitates the incorporation of additives and does not ensure their complete removal, which may potentially compromise the stability of the devices. Here, a simple additive‐free in situ surface polishing (ISSP) strategy is proposed to reconstruct the surface of perovskite films and mitigate *V_OC_* loss. The ISSP treatment, based on a green polishing agent of 1,1,1,3,3,3‐hexafluoropropan‐2‐ol (HFIP), has demonstrated its effectiveness in reducing surface defects, optimizing energy level alignment, and improving interfacial contacts. The ISSP‐treated PSCs exhibit a minimal *V_OC_* loss of 0.35 V, approaching 95% of the theoretical *V_OC_* limit. As a result, the PSCs exhibit high power conversion efficiency (PCE) and stability, with a high PCE of 24.13% and maintaining 91.1% of the initial PCE after over 2,500 h of N_2_ storage (ISOS‐D‐1). Furthermore, the ISSP treatment is applicable to flexible PSCs (FPSCs) to obtain both high efficiency and mechanical robustness.

## Graphical abstract

A novel additive‐free in situ surface polishing (ISSP) strategy is proposed to reconstruct the perovskite surface and mitigate open‐ circuit voltage *V_OC_* loss. The ISSP treatment effectively passivates the surface defects as well as improves both interfacial contact and energy level alignment. The ISSP‐treated perovskite solar cells (PSCs) exhibit a minimal *V_OC_* loss of 0.35 eV, approaching 95% of the theoretical limit with a power conversion efficiency (PCE) of 24.13%.

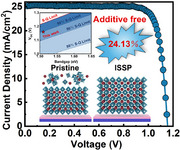

## 1 Introduction

Perovskite materials have garnered significant interest in the field of optoelectronics, including photovoltaics, light‐emitting diodes (LEDs), sensors, and photodetectors, due to their excellent light absorption capability, high carrier mobility, and adjustable bandgap.^[^ ^1^, ^2^, ^3^, ^4^, ^5^, ^6^, ^7^, ^8^ ^]^ Perovskite solar cells (PSCs), in particular, have achieved a noteworthy power conversion efficiency (PCE) of 27.0% in the past decade, making them a promising alternative to crystalline silicon solar cells.^[^ ^9^ ^]^ Despite the short‐circuit current (*J_SC_*) has essentially reached the theoretical limit, there is still a certain gap between the open‐circuit voltage (*V_OC_*) and the theoretical limit.^[^ ^10^, ^11^, ^12^ ^]^ It is therefore essential to further enhance the *V_OC_* to approach the theoretical limit and thereby facilitate further improvements in the PCE of PSCs. Polycrystalline perovskite films often contain numerous defects due to their ionic nature, and the undesired defects act as nonradiative recombination centers, leading to a notable *V_OC_* loss, thus severely affecting the photovoltaic performance and stability of the devices.^[^ ^13^, ^14^, ^15^, ^16^, ^17^, ^18^ ^]^ Given that surface defects are two orders of magnitude larger than the bulk of the perovskite films, targeted modification of surface defects is essential to mitigate the *V_OC_* loss.^[^ ^19^, ^20^ ^]^

Considerable efforts have been made to passivate surface defects of the perovskite films using chemical additives, such as small molecules and polymers.^[^ ^21^, ^22^, ^23^, ^24^, ^25^ ^]^ Although those approaches have led to notable improvements in *V_OC_* and PCE of PSCs, several challenges remain, including the complexity of the process, the incompatibility of solvents with the underlying crystalline regions, the specificity of additives to certain perovskite components and the risk of secondary contamination from the excessive additives.^[^ ^26^, ^27^ ^]^ Polishing has recently emerged as an effective alternative to conventional chemical additive modification by removing the surface defects.^[^ ^28^, ^29^, ^30^, ^31^, ^32^ ^]^ For instance, Huang et al. demonstrated that the perovskite surface was mechanically soft and poorly bonded to the underlying crystalline regions, making it easy to remove the nanocrystals and amorphous regions on the perovskite surface with adhesive tapes.^[^ ^28^ ^]^ Xie et al. proposed a “clean passivation” method by using a multifunctional amino acid salt (S)‐3‐amino‐4‐phenylbutyric acid hydrochloride (ApaCl) to passivate surface defects, followed by washing away the passivator residue, achieving a high PCE of 24.27% and a notable reduction in the *V_OC_* loss by 90 mV.^[^ ^31^ ^]^ Saliba et al. demonstrated a solvent‐free, automated, and potentially scalable method of polishing the perovskite surface by a non‐contact nanosecond pulsed ultraviolet laser, resulting in a significant improvement in the *V_OC_* from 1.08 to 1.14 V.^[^ ^32^ ^]^ However, most of the current polishing methods applied for PSCs require the use of additional additives as well as complex removal steps. Developing an additive‐free, universal, and effective polishing method to mitigate the *V_OC_* loss is still challenging.

Here, a simple and universal one‐step strategy is proposed for reconstructing the surface of perovskite films and mitigating the *V_OC_* loss without undesired residuals. The perovskite surface containing nanocrystals and amorphous regions, is polished in situ by a green solvent of 1,1,1,3,3,3‐hexafluoropropan‐2‐ol (HFIP) as the polishing agent. HFIP is considered as an ideal solvent with high dissolving power and environmentally friendly characteristics and has been widely employed as a hydrogen bond donor in the preparation of hydrophobic deep eutectic solvents.^[^ ^33^ ^]^ The in situ surface polishing (ISSP) treatment significantly eliminates surface defects and improves carrier transport. As a result, the ISSP‐treated PSCs with the favorable perovskite surface achieve a PCE of 24.13%, with a minimal *V_OC_* loss of 0.35 V, which approaches 95% of the theoretical *V_OC_* limit. The ISSP‐treated devices have demonstrated good stability by maintaining 91.1% of the initial PCE after over 2500 h of N_2_ storage (ISOS‐D‐1). The ISSP treatment has been further applied to flexible PSCs (FPSCs) to obtain an excellent PCE of 22.14% and mechanical robustness with 81.9% of the initial PCE undergoing over 5000 bending cycles with a bending radius of 4 mm.

## 2 Results and Discussion

**Figure 1:**
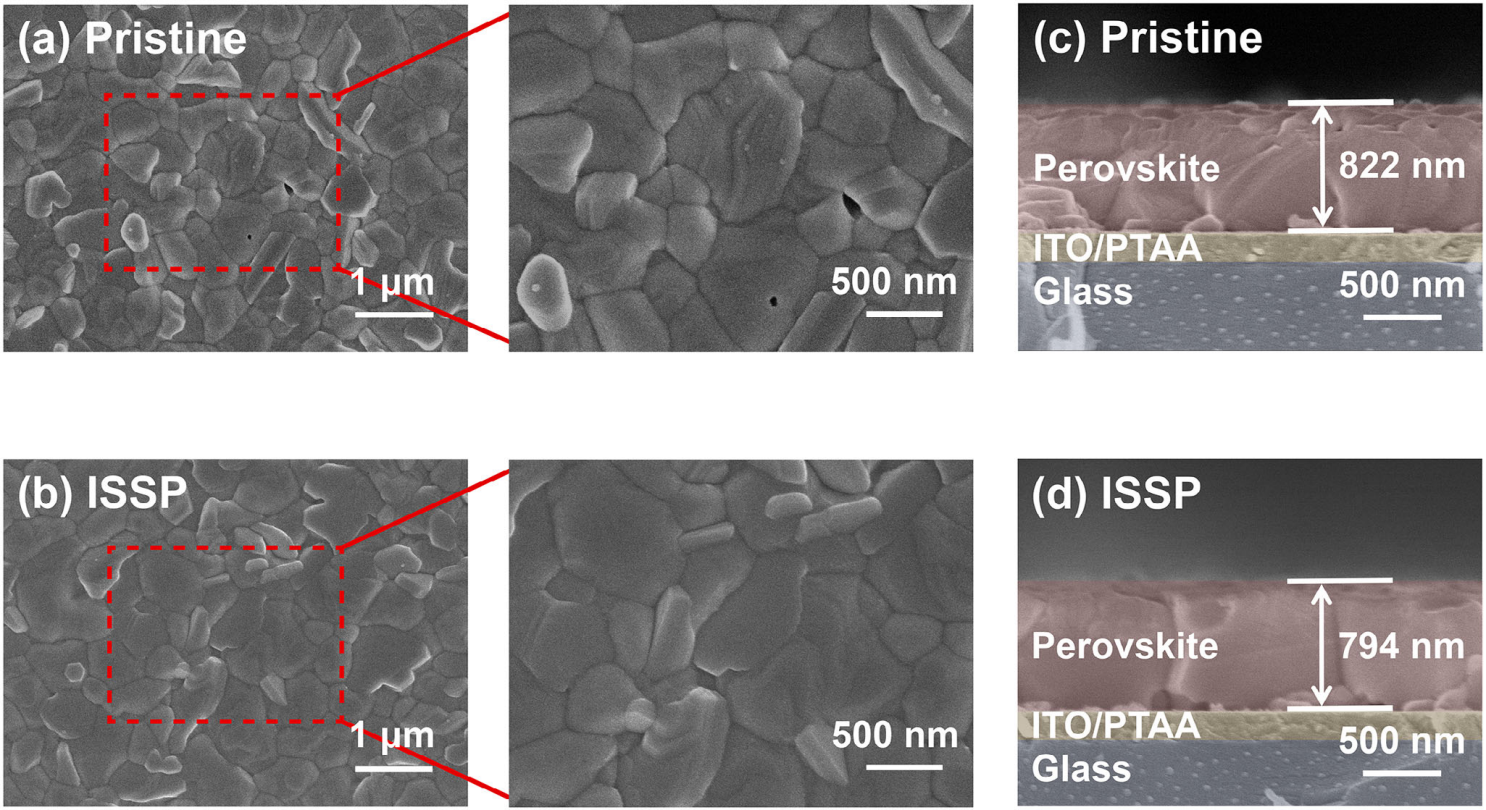
Morphological characterizations of the perovskite films. Top‐view SEM images of a) pristine and b) ISSP‐treated perovskite films. Cross‐sectional SEM images of c) pristine and d) ISSP‐treated perovskite films.

The chemical structure of the proposed ISSP agent of HFIP is shown in Figure S1 (Supporting Information). HFIP has high polarity and exhibits high solubility for perovskite. The HFIP makes brief contact with the surface of the perovskite films by a dynamic spin‐coating method, thereby polishing the surface defect regions. Meanwhile, the HFIP that remains on the surface of the perovskite film would dissolve the microcrystals on the surface of the perovskite and promote secondary crystal growth to reconstruct the surface of the perovskite films during the thermal annealing process (Figure S2, Supporting Information).^[^ ^34^, ^35^ ^]^ The scanning electron microscope (SEM) images in **Figure** **1** clearly illustrate the difference in surface morphology of the perovskite films before and after ISSP treatment. The pristine perovskite film contains some voids on the surface (Figure 1a), which may increase the leakage current in PSCs. In contrast, the ISSP‐treated perovskite films are more uniform, denser, pinhole‐free, and have larger grain sizes, which is attributed to the effective polishing of defective surface regions of perovskite films by HFIP and the secondary crystal growth of the perovskite films during the thermal annealing process (Figure 1b; S3, Supporting Information). The grain boundaries of the perovskite films are enriched with deep‐level defects, which would act as the non‐radiative recombination centers to trap carriers, thereby affecting the transport of carriers.^[^ ^36^, ^37^ ^]^ The ISSP‐treated perovskite films have larger grain sizes and fewer grain boundaries, which are favorable for carrier transport, and thus better device performance is predictable. The cross‐sectional SEM in Figure 1c,d reflects the slight decrease in thickness of the perovskite films from 822 to 794 nm after ISSP treatment, demonstrating that the polishing treatment has a removal effect on the defective surface regions.

The surface properties of the ISSP‐treated perovskite films were further investigated by atomic force microscopy (AFM). The root mean square (RMS) is decreased significantly from 15.4 to 10.2 nm by the ISSP treatment (Figure S4a,b, Supporting Information), demonstrating that the ISSP process provides a flat and homogeneous perovskite surface, which could promote the closely contact with the electron transport layer (ETL) and facilitate the carrier transport in PSCs. Moreover, the 3D AFM images in Figure S4c,d (Supporting Information) demonstrate more visually that the reduced surface undulations of perovskite films following ISSP treatment.

**Figure 2:**
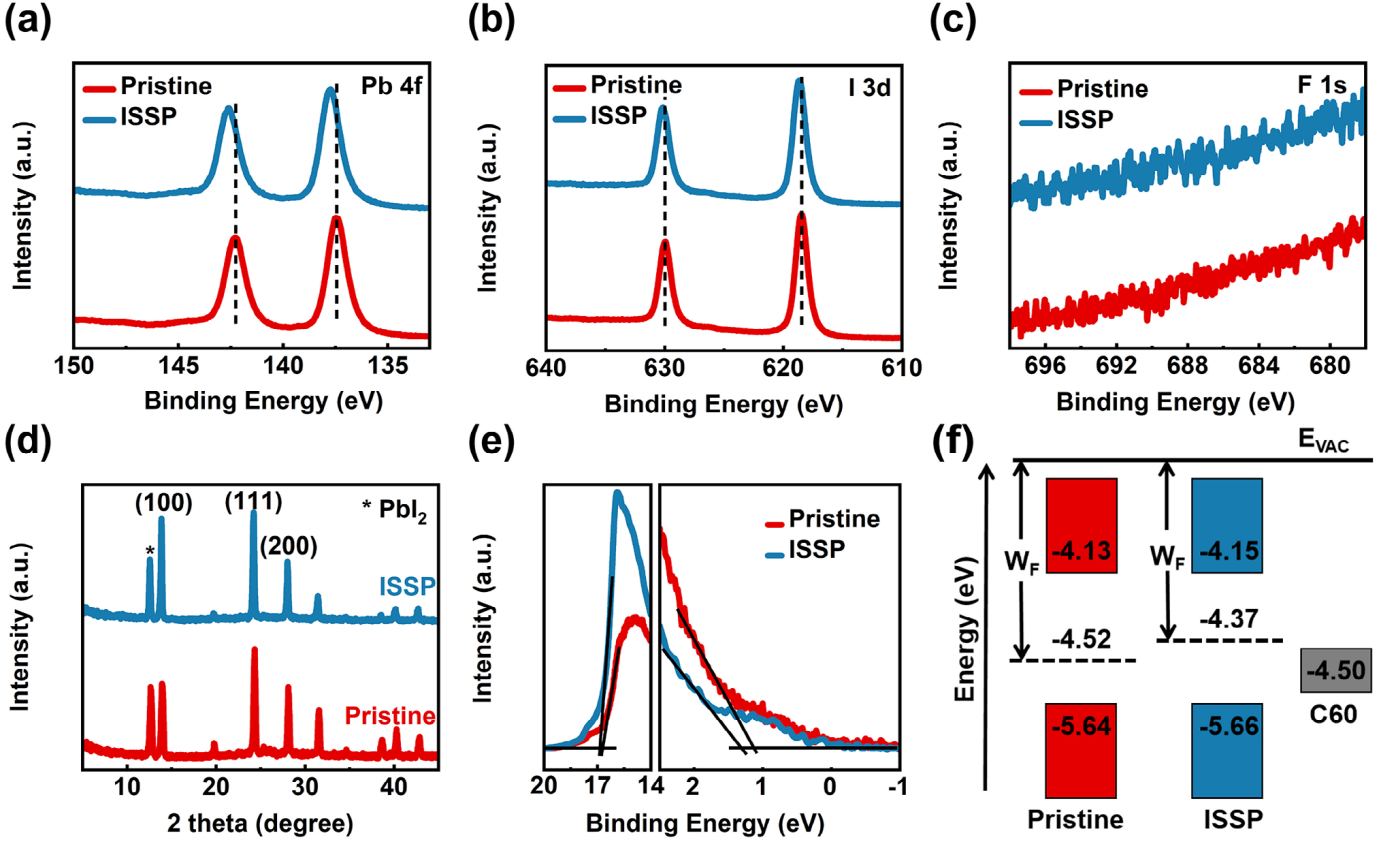
Structural characterizations of the perovskite films. XPS spectra of a) *Pb 4f*, b) *I 3d*, and c) *F 1s* spectra of the pristine and ISSP‐treated perovskite films. d) XRD patterns, e) UPS spectra, and f) energy‐level diagram of the pristine and ISSP‐treated perovskite films.

X‐ray photoelectron spectroscopy (XPS) was performed to characterize the structure properties of the ISSP‐treated perovskite films (**Figure** **2**a–c). As shown in Figure 2a,b, the binding energies of the *Pb 4f* and *I 3d* core levels of the ISSP‐treated samples exhibit a slight shift toward higher energy in comparison to the pristine films, indicating the enhanced binding of I^−^ and Pb^2+^ on the surface of the perovskite films. HFIP can effectively remove the loose perovskite surface regions while having minimal effect on PbI_2_ due to the low solubility of HFIP for PbI_2_. Furthermore, the binding energies of Pb and I in pure PbI_2_ are much stronger than perovskite.^[^ ^34^ ^]^ Consequently, the increased binding energies of *Pb 4f* and *I 3d* core levels can be attributed to the slight excess of PbI_2_ on the surface of the perovskite films by ISSP treatment. The identical *F 1s* core levels indicate a clean perovskite surface without residuals of the polishing agent HFIP after the ISSP process (Figure 2c). Additionally, the energy dispersive X‐ray spectroscopy (EDS) results further verify the “residue‐free” property of the ISSP treatment, which is consistent with the XPS results (Figure S5, Supporting Information). X‐ray diffraction (XRD) patterns in Figure 2d exhibit a stronger (100) diffraction peak, while the position remains unchanged, implying the promoted secondary growth of perovskite crystals.^[^ ^34^ ^]^ Meanwhile, the ISSP‐treated perovskite films exhibited identical characteristic peaks to those of the pristine films, indicating that ISSP treatment does not damage the crystal structure of perovskite. Furthermore, no new diffraction peaks are found, demonstrating that the ISSP treatment would not introduce any new crystalline phases or extrinsic components in the final perovskite film, which is consistent with the XPS results. The relatively high (100) diffraction peak of PbI_2_, located at 12.7°, can be attributed to the incomplete solid‐liquid reaction. In the two‐step sequential deposition method, the pre‐prepared PbI_2_ film undergoes a solid‐liquid reaction with organic salts, resulting in the final transformation into the perovskite phase.^[^ ^38^ ^]^ However, the dense PbI_2_ film would hinder the effective penetration of organic salts and the solid‐liquid reaction, which results in the presence of residual PbI_2_ in the resulting perovskite films.^[^ ^39^, ^40^ ^]^ The UV–vis absorption spectra of perovskite films are compared in Figure S6a (Supporting Information), a slightly improved absorption in the visible range after ISSP treatment can be observed because of the enhanced crystallinity. Figure S6b (Supporting Information) shows the corresponding tauc plots and exhibits a negligible change on the band gap, which was measured both to be 1.51 eV for the pristine and ISSP‐treated perovskite films.

Surface defects in perovskite films could act as nonradiative recombination centers and also impact the carrier transports, which significantly limit the *V_OC_* of PSCs.^[^ ^41^ ^]^ Ultraviolet photoelectron spectroscopy (UPS) measurements were used to explore the interfacial energy alignment of perovskite films induced by ISSP (Figure 2e). The conduction band (*E_C_*) and valence band (*E_V_*) values were calculated according to the reported method,^[^ ^42^ ^]^ and the detailed electronic energy levels are listed in Table S1 (Supporting Information). The corresponding energy level diagram of perovskite films is shown in Figure 2f. Compared to the pristine film, the ISSP‐treated film exhibits an upshift of the work function (W_F_) by 0.15 eV, resulting in a more n‐type surface. The removal of the defective surface by ISSP treatment results in a change in the surface termination, which in turn results in a shift in the surface energy level. Better matched energy levels between the perovskite layer and ETL (C60) in PSCs have been achieved, thereby facilitating the efficient carrier transport.

**Figure 3:**
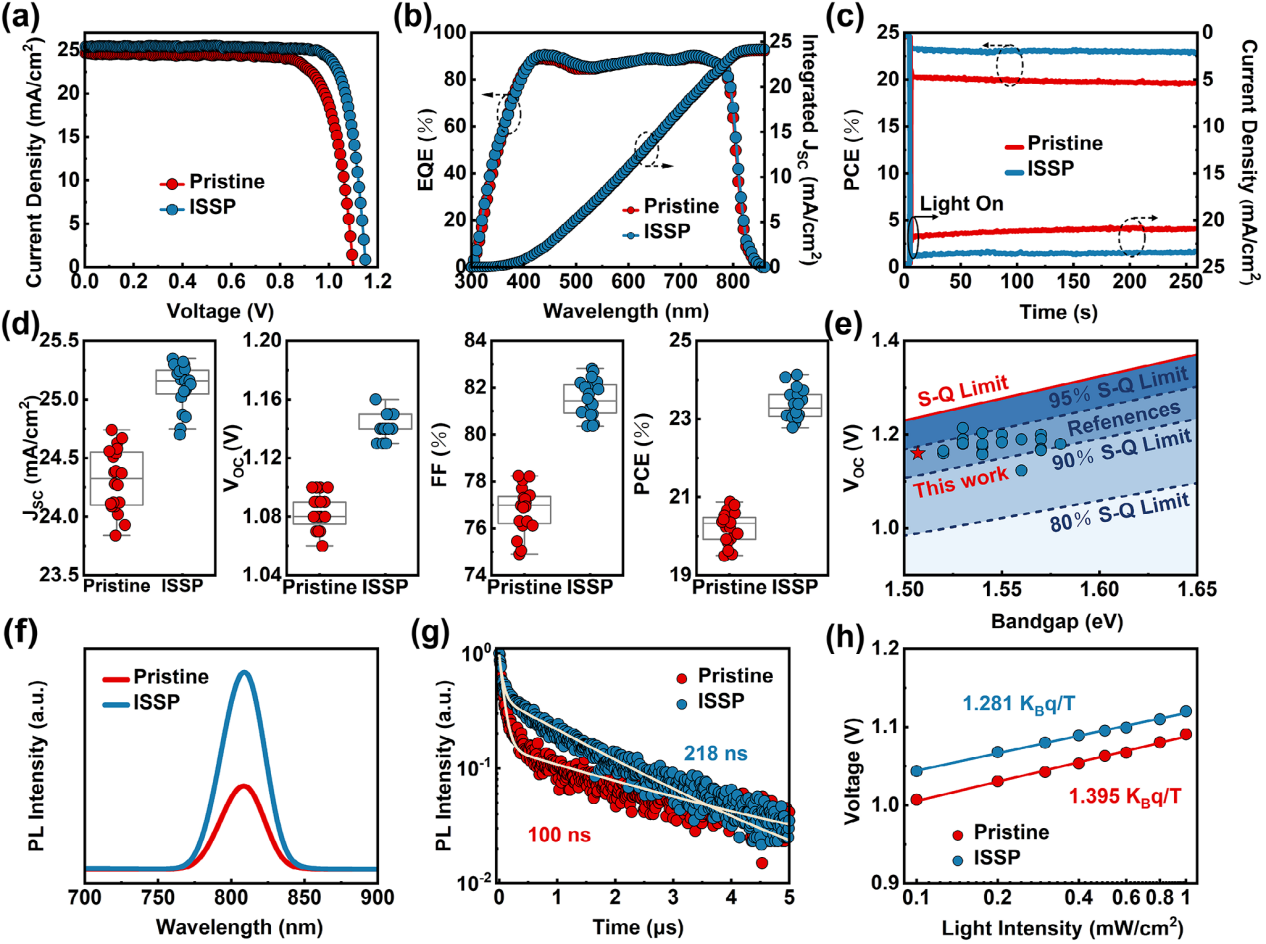
Photovoltaic performance of the PSCs. a) *J‐V* curves of the optimal PSCs. b) EQE spectra of the PSCs. c) SPO measurements of the PSCs. d) Statistics distribution of the *V_OC_*, *J_SC_*, *FF*, and PCE of the PSCs. e) *V_OC_* comparison of PSCs with bandgap narrower than 1.6 eV. f) PL spectra and g) TRPL decay spectra of the pristine and ISSP‐treated perovskite films. (The perovskite films are deposited on the glass substances.) h) The dependence of *V_OC_* on the light intensity of the PSCs.

As discussed above, the proposed ISSP treatment based on the polishing agent HFIP can modulate both film morphology and interfacial energy alignment. We applied the ISSP treatment in PSCs to regulate the photovoltaic performance. As shown in Figure S7 (Supporting Information), the inverted PSCs were prepared with a structure of glass/ITO/PTAA/FA_0.92_MA_0.08_PbI_3_/C60/BCP/Cu. The optimal volume of HFIP for ISSP treatment was determined by gradually increasing its volume from 35 to 140 µL. The PCE of the devices increases as the volume of HFIP increases, and the highest PCE with significantly improved *V_OC_* and *FF* is achieved when 70 µL of HFIP is applied. However, further increasing the volume of HFIP leads to a decrease in PCE, which is mainly due to the damage of crystalline regions by excessive polishing, resulting in a decrease in *FF* (Figure S8 and Table S2, Supporting Information). As shown in **Figure** **3**a and **Table** 1, the optimal pristine device demonstrates a PCE of 20.88%, while the optimal ISSP‐treated device delivers a much higher PCE of 24.13%, which is among the best PCE of PSCs based on polishing treatment (Table S3, Supporting Information). The hysteresis index (HI) markedly decreases from 2.72% to 0.52% by ISSP treatment, which can be attributed to the improved surface properties of the perovskite (Figure S9 and Table S4, Supporting Information).^[^ ^43^ ^]^ The integrated photocurrent density obtained from the external quantum efficiency (EQE) spectra of the optimal pristine and ISSP‐treated PSCs is 23.97 mA cm^−2^ and 24.21 mA cm^−2^, respectively, which is consistent with the *J–V* performance (Figure 3b). Furthermore, the steady‐state power output (SPO) measurements were conducted (Figure 3c). The ISSP‐treated PSC exhibits a stable PCE of 23.5% at the bias of 0.98 V under continuous AM 1.5 illumination for 250 s, while the pristine PSC shows a much lower stable PCE of 19.9%, which fit in well with the results obtained from the *J–V* curves. Statistics distributions of photovoltaic parameters are shown in Figure 3d and detailed parameters are summarized in Tables S5 and S6 (Supporting Information). The *V_OC_* and *FF* exhibit general increases, which account for the significant enhancement in PCE, demonstrating the good reproducibility of ISSP treatment.

The *V_OC_* loss is markedly diminished, with the highest *V_OC_* of 1.16 V being achieved, approaching 95% of the theoretical limit for a 1.51 eV bandgap perovskite absorber (Figure 3e; Table S6, Supporting Information). To further quantify the effect of the ISSP treatment on the *V_OC_* loss, the highly sensitive EQE and the electroluminescent (EL) spectra are conducted and each part of the *V_OC_* loss is calculated based on the detailed balance theory (Figure S10, Supporting Information).^[^ ^44^ ^]^ Compared with the pristine devices, the *ΔV_2_* and *ΔV_3_* of the ISSP‐treated devices are significantly reduced, indicating that the ISSP treatment can effectively remove the defective surface regions and improve the quality of the perovskite films, thus suppressing the non‐radiative recombination loss, which is consistent with the *J–V* results.

To investigate the impact of ISSP treatment on the surface defects of the perovskites, steady‐state photoluminescence (PL) and time‐resolved PL (TRPL) spectra were conducted. As illustrated in Figure 3f, the significantly enhanced PL intensity is obtained by ISSP treatment. Furthermore, the ISSP‐treated perovskite film exhibits a much longer average carrier lifetime (*τ_ave_*) of 218 ns in comparison to the pristine sample of 100 ns (Figure 3g; Table S8, Supporting Information). These results suggest that defect‐induced nonradiative recombination are suppressed by ISSP treatment.^[^ ^45^, ^46^, ^47^ ^]^ To quantify the defects of perovskite films, space charge limiting current (SCLC) measurements were performed to calculate the defect density (*N_trap_*) in Figure S11 (Supporting Information) and Supplementary Note 1. The significantly lowered *N_trap_* further indicates that ISSP treatment can effectively remove surface defects, thus suppressing nonradiative recombination, and increased *V_OC_* and *FF* could be expected in ISSP‐treated PSCs. The dependency of *V_OC_* on log‐scaled illumination intensity is shown in Figure 3h. The slope of the ISSP‐treated PSCs (1.281 k_B_T/q), which is associated with the ideal factor to identify the dominant recombination mechanism, is closer to 1 compared to the pristine device (1.395 k_B_T/q), indicating a significant inhibition of trap‐assisted nonradiative recombination.^[^ ^48^ ^]^ The lowered leakage current provides further evidence that improved charge transport and suppressed nonradiative recombination by ISSP treatment (Figure S12, Supporting Information). In addition, the short charge extraction time (*τ_t_*) of ISSP‐treated film obtained from the transient photocurrent (TPC) spectra also demonstrates the improved carrier transport at the perovskite interface due to the effective removal of surface defects and improved band alignment by the proposed ISSP treatment (Figure S13, Supporting Information).^[^ ^49^ ^]^

We further compared the *J–V* performances of inverted PSCs based on various commonly used post‐treatment solvents for ISSP treatment to verify the uniqueness of HFIP (Figure S14, Supporting Information), and the detailed parameters are summarized in Table S9 (Supporting Information). The PCE remains almost unchanged after surface polishing by toluene (TL) and chlorobenzene (CB), respectively. In contrast, the isopropanol (IPA) and HFIP‐treated PSCs demonstrate enhanced PCE in comparison to the pristine device, while the HFIP‐treated PSCs exhibit significantly higher *V_OC_* and PCE. The presence of fluorine atoms with a high electronegativity in the HFIP molecule results in a strong attraction to electrons, thereby reducing the electron density of the carbon atoms connected to the hydroxyl group, resulting in the electron cloud of the hydroxyl being more biased in favor of the oxygen atoms. Consequently, the hydrogen atoms are more positively charged and more likely to act as a hydrogen bonding donor for the formation of hydrogen bonding with other atoms or groups with a lone pair of electrons.^[^ ^50^ ^]^ Furthermore, HFIP exhibits higher polarity than IPA due to the presence of strongly polar fluorine atoms within the molecule (Table S10, Supporting Information). Based on the “like‐dissolves‐like” rule, solvents with higher polarity guarantee the better solubility for the FA‐based perovskite with high polarity,^[^ ^51^ ^]^ and the polishing agent with lower polarity, such as TL or CB, cannot effectively remove the surface defects of perovskite.^[^ ^34^ ^]^ As a result, the proposed polishing agent of HFIP, with the strong hydrogen bond donor capability and high polarity, provides the best improvement in photovoltaic performance by effectively dissolving and polishing the defective surface regions of the perovskite films. We further applied ISSP treatment with HFIP to various perovskite compositions, as shown in Figures S15, S16, and Tables S11, S12 (Supporting Information), and PCE of PSCs are enhanced to different degrees after ISSP treatment, implying the effectivity, applicability, and universality of the proposed ISSP strategy based on HFIP in PSCs.

**Figure 4:**
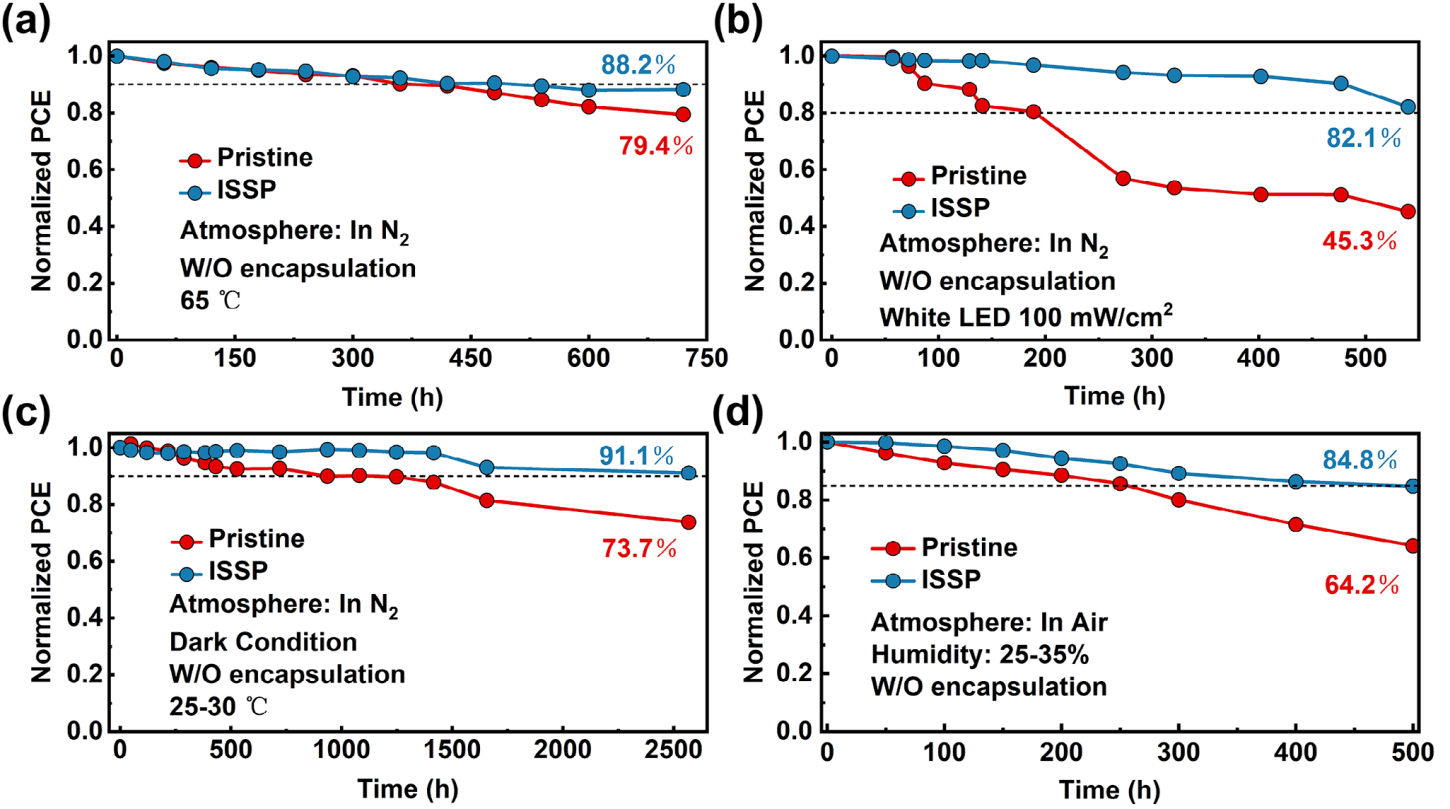
Stability of the PSCs. a) Normalized PCE of the unencapsulated PSCs at a fixed temperature of 65 °C in a N_2_ atmosphere (ISOS‐L‐1). b) Normalized PCE of the unencapsulated PSCs under continuous light soaking with a white LED lamp, 100 mW cm^−2^ in a N_2_ atmosphere (ISOS‐D‐2). c) Normalized PCE of the unencapsulated PSCs storage in a N_2_ atmosphere (ISOS‐D‐1). d) Normalized PCE of the unencapsulated PSCs stored in an ambient environment (ISOS‐D‐1).

To investigate the impact of ISSP treatment on device stability, the PCE changes of the unencapsulated PSCs were examined under the International Summit on Organic Photovoltaic Stability (ISOS) protocols.^[^ ^52^ ^]^ The heat stability of the devices was monitored under a N_2_ atmosphere at 65 °C (ISOS‐L‐1). **Figure** **4**a shows that the ISSP‐treated device can maintain 88.2% of the initial PCE for 720 h, while the pristine device drops to 79.4%. The light stability was measured by subjecting devices to continuous white LED light soaking (100 mW/cm^2^) for 540 h under a N_2_ atmosphere, and the ISSP‐treated device exhibited the 82.1% of the initial PCE, while the pristine device significantly decreases to 45.3% (ISOS‐D‐2) (Figure 4b). For long‐term stability, the ISSP‐treated device demonstrates a *T91* (time to 91% of initial PCE) of over 2500 h in a N_2_ environment, while the pristine device only retains 73.7% of the initial performance (ISOS‐D‐1) (Figure 4c). The ISSP‐treated device also exhibited enhanced ambient stability, with 84.8% of the initial PCE retained after being stored in an ambient environment (relative humidity (RH) of 25–35%) for 500 h (ISOS‐D‐1) (Figure 4d). In contrast, the pristine device demonstrated a rapid decline, reaching only 64.2% of the initial PCE. Furthermore, the encapsulated devices were aged in humid air environment (RH of 50–65%). As shown in Figure S17 (Supporting Information), the ISSP‐treated device can maintain 80.7% of the initial PCE, while the pristine device dropped to 54.2%, which is consistent with the above results. The enhanced stability is directly linked to the improved surface properties. Typically, degradation occurs at interfaces and grain boundaries of perovskite with defect enrichment, and the defect sites act as nonradiative recombination centers leading to a significant decrease in device performance.^[^ ^53^ ^]^ The ISSP treatment reconstructs the surface of the perovskite layer by effectively removing nanocrystals and amorphous regions, resulting in a significant improvement in device stability by reducing the pathways of degradation.

**Figure 5:**
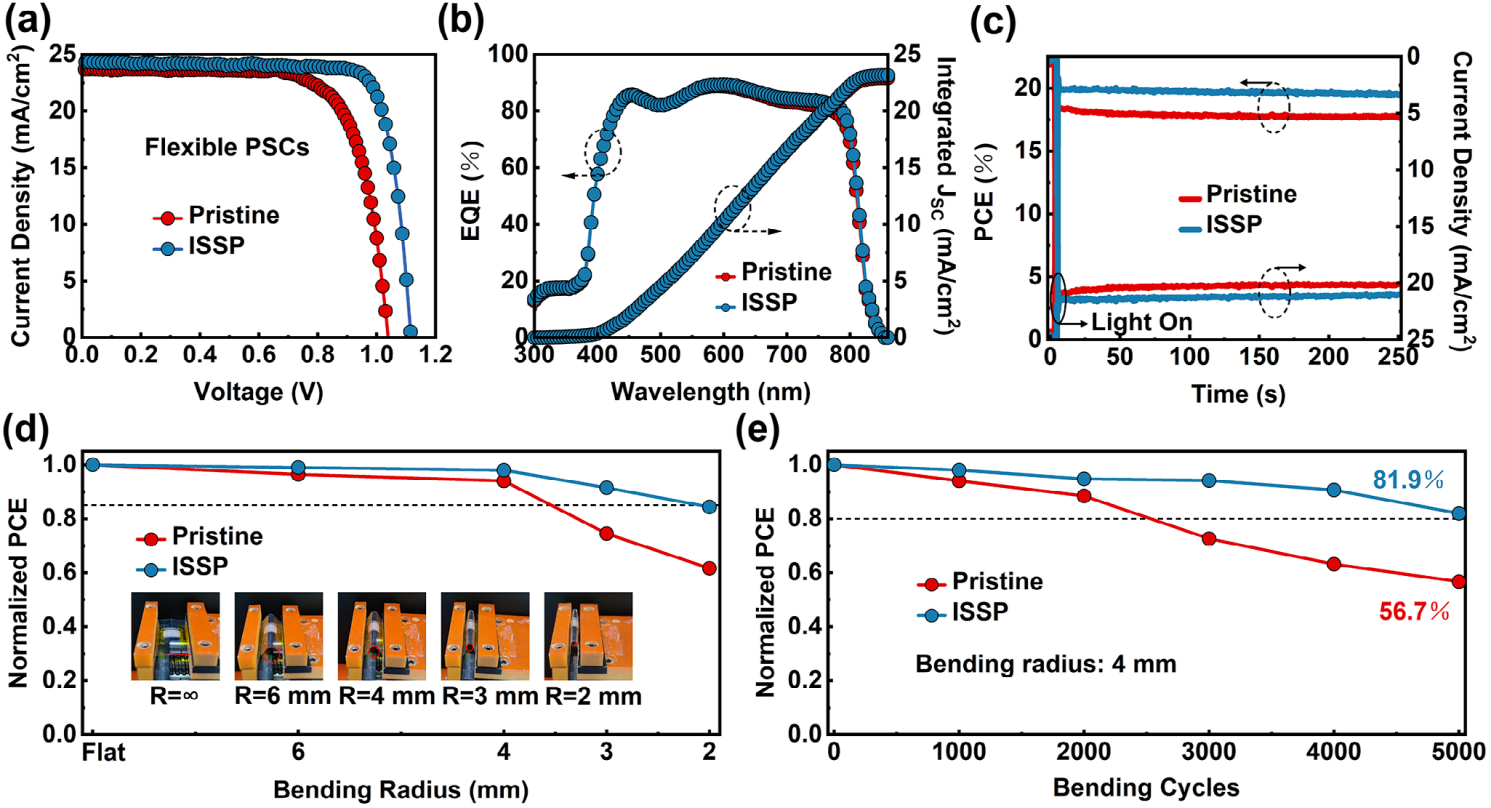
Photovoltaic performance and mechanical stability of the FPSCs. a) *J–V* curves of the optimal FPSCs. b) EQE spectra of the FPSCs. c) SPO curves of the FPSCs. d) Normalized PCE of the unencapsulated FPSCs as a function of bending radius with bending cycles of 1000 times. e) Normalized PCE of the unencapsulated FPSCs as a function of bending cycles with a bending radius of 4 mm.

The ISSP treatment is applicable to FPSCs to improve their performances. The FPSCs were fabricated on PEN/ITO flexible substrates with the same device construction to the rigid PSCs. The optimal ISSP‐treated FPSCs demonstrate an excellent PCE of 22.14%, which far exceeds that of the pristine counterpart (18.04%), as shown in **Figure** **5**a and Table S13 (Supporting Information). The integrated photocurrent densities of the FPSCs reach 22.93 and 23.16 mA cm^−2^, respectively, which is consistent with the *J‐V* results (Figure 5b). The variation in *J_SC_* between flexible and rigid devices is mainly due to the difference in transmittance between the PEN/ITO substrate and the glass/ITO substrate as compared in Figure S18 (Supporting Information). In additional, the ISSP‐treated device provides a more stable and higher steady‐state output near the maximum power point (MPP) under continuous 1 sun illumination for 250 s without attenuation compared to the pristine device (Figure 5c).

Mechanical stability is a critical property for the application of FPSCs. Repeated bending tests with 1000 bending cycles and varying bending radius has been conducted on FPSCs (Figure 5d). The ISSP‐treated FPSCs show improved mechanical stability. At a small bending radius of 2 mm, the ISSP‐treated device can maintain 81.4% of the initial performance, while the pristine device decays to 61.6%. The multi‐cycle bending test with a fixed bending radius of 4 mm was further applied. Figure 5e shows that the ISSP‐treated device is more robust, maintaining 81.9% of the initial PCE even after 5000 bending cycles, while the pristine device decreases significantly to 56.7%. The ISSP treatment effectively removes the loose and defective surface, and the surface reconstruction induced by ISSP increases the grain size and reduces surface roughness, which improves the contact between the perovskite layer and the ETL with an enhanced interfacial toughness, leading to the mechanical robustness of the ISSP‐treated devices.

**Table 1:** The *J‐V* parameters of the optimal pristine and ISSP‐treated PSCs.

Sample	*V_OC_* (V)	*J_SC_* (mA cm^−2^)	*FF* (%)	PCE (%)
Pristine	1.10	24.63	77.06	20.88
ISSP‐treated	1.15	25.30	82.82	24.13

## 3 Conclusion

In summary, we propose an ISSP treatment with the green polishing agent of HFIP to reconstruct the defective surface of perovskite and mitigate the *V_OC_* loss. The high polarity of HFIP and the appropriate process allow for the effective removal of the nanocrystals and amorphous defective surface without destroying the underlying crystalline regions or leaving any residuals. Simultaneously, a more n‐type surface is formed, which facilitates the carrier transport in PSCs. The inverted PSCs have demonstrated a notable improvement in both *V_OC_* and *FF* due to the improved surface properties based on the proposed ISSP treatment, leading to a high PCE of 24.13%. Meanwhile, an extremely high *V_OC_* of 1.16 V is obtained, which approaches 95% of the theoretical limit for a 1.51 eV bandgap perovskite absorber. By reducing the pathways of degradation, the ISSP‐treated devices show dramatically improved stability by maintaining 91.1% of the initial PCE after over 2500 h of N_2_ storage. The ISSP‐treated FPSCs also demonstrate high photovoltaic performance and excellent mechanical robustness with minor degradation in PCE after 5000 bending cycles with a bending radius of 4 mm. The proposed ISSP treatment is a universal and effective performance improvement strategy for a wide range of perovskite compositions, which would broaden the applications of the perovskites in various optoelectronics.

## Conflict of Interest

The authors declare no conflict of interest.

## Supporting information



Supporting Information

## Data Availability

The data that support the findings of this study are available from the corresponding author upon reasonable request.
