# Supplementary material for: In situ Surface Polishing Enables VOC Approaching 95% of the Theoretical Limit for Efficient Inverted Perovskite Solar Cells
Source: Adv Sci (Weinh). 2025 Mar 31;12(26):2503342. doi: 10.1002/advs.202503342 (PMC12245114; doi:10.1002/advs.202503342)
Supplement: Supplementary file 1 — Supporting Information [file ADVS-12-2503342-s001.docx]

Supporting Information

In situ Surface Polishing Enables *V_OC_* Approaching 95% of the Theoretical Limit for Efficient Inverted Perovskite Solar Cells

*Han-Wen Zhang,^a^ Yan-Gang Bi,^a^* Yi-Fan Wang,^a^ Mu Lin,^a^ Chang Liu^a^ and Jing Feng^a^**

a State Key Laboratory of Integrated Optoelectronics, College of Electronic Science and Engineering, Jilin University, 2699 Qianjin Street, Changchun 130012, China.

* Corresponding authors

**Experimental Section**

**Materials:**

Formamidinium iodide (FAI, 99.5%), methylammonium iodide (MAI, 99.5%), Methylammonium chloride (MACl, 99.5%) and Lead(II) iodide (PbI_2_, 99.99%) were purchased from Advanced Election Technology Co., Ltd. Fullerene C60 and 2,9-dimethyl-4,7-diphenyl-1,10-phenanthroline (BCP) were purchased from Luminescence Technology Co. Poly[bis(4-phenyl) (2,4,6-trimethylphenyl) amine (PTAA) was purchased from Xi'an Yuri Solar Co., Ltd. The anhydrous solvents N,N-dinbmethylformamide (DMF, 99.8%), dimethyl sulfoxide (DMSO, ≥99.9%), toluene (TL, 99.8%), Chlorobenzene (CB, 99.8%) and isopropanol (IPA, ≥99.7%) were purchased from Sigma-Aldrich. 1,1,1,3,3,3-hexafluoropropan-2-ol (HFIP, >99.5%) was purchased from TCI Development Co., Ltd. All materials were received and used without any purification.

**Device Fabrication:**

Patterned glass/ITO substances were cleaned by washing with acetone and IPA for 30 mins, and patterned PEN/ITO substances were cleaned by washing with IPA for 30 mins prior to use and then dried by N_2_ blowing. The substrates were further exposed to ultraviolet ozone treatment for 10 mins before being transferred to the N_2_-filled glove box for the following spin coating procedures. PTAA as the hole transport layer was dissolved in TL (2 mg/mL) and spin-coated onto the substrates at 5,000 rpm for 30 s followed by annealing at 100 ℃ for 10 mins. Then 80 µL of PbI_2_ precursor was spin-coated (1.5 M PbI_2_ in DMF: DMSO at a volume ratio of 9: 1) onto the substrates at 1,500 rpm for 30 s and annealed at 70 ℃ for 60 s in the N_2_-filled glove box. After cooling down to room temperature, the organic salt solution prepared by dissolving FAI (90.0 mg), MAI (6.4 mg) and MACl (9.0 mg) in IPA solvent (1 mL) was spin-coated onto the PbI_2_ film at 2,500 rpm for 30 s and followed by annealing at 150 ℃ for 15 mins in air with 20-30% relative humidity (RH). For surface polishing process, different surface polishing agent were dynamically spin-coated on the perovskite surface at 4000 rpm for 30 s and then annealed at 100 ℃ for 5 mins in the N_2_-filled glove box. Finally, C60 (20 nm), BCP (6 nm), and Cu electrode (100 nm) were deposited at a rate of 0.3, 0.2 and 1.0 Å/s upon perovskite films by thermal evaporation.

**Characterization:**

The *J-V* curves of the PSCs were measured by a Keithley 2400 source meter, under AM 1.5G irradiation with irradiance uniformity, temporal instability of irradiance and spectral match (AAA grade) solar simulator (XES-70S1, SAN-EI Electric co., Ltd.) in an N_2_-filled glove box. The *J-V* hysteresis was obtained by forward (from -0.2 V to 1.2 V) and reverse scans (from 1.2 V to -0.2 V) at a scan rate of 100 mV/s. A standard Si solar cell equipped with a KG-5 filter was applied to calibrate the light intensity (100 mW/cm^2^). EQE was detected by a QE-R3011 system (Enli Technology co., Ltd.). The roughness of the perovskite was scaled by atomic force microscope (AFM, Dimension Icon). The surface profile of the perovskite was characterized by using a scanning electron microscope (SEM, JSM-7500F). The UV-vis absorption spectra were measured using a UV-vis diffuse reflectance spectrophotometer (Hatachi U3900H). The XRD measurement was obtained using a Rigaku D/max 2550 X-ray diffractometer with a monochromatic Cu target radiation source.


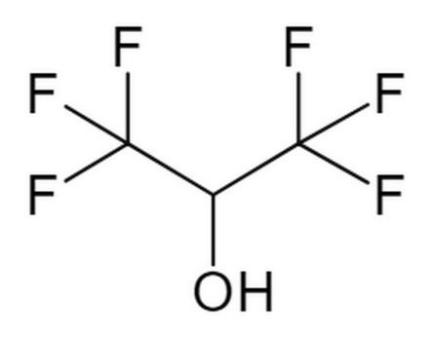
**Figure S1.** Chemical structure of HFIP.

**Figure
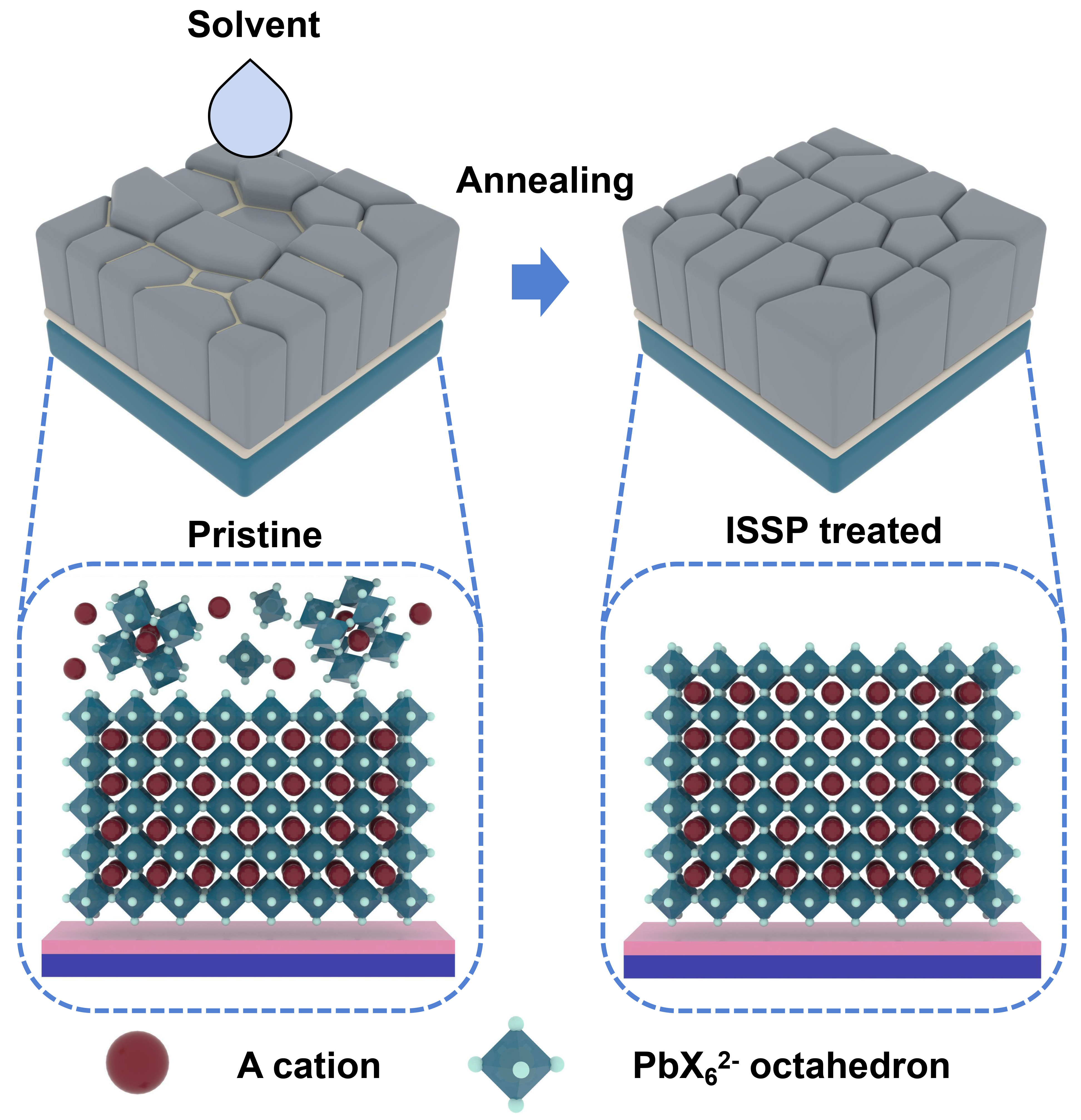
 S2.** Schematic illustration of the ISSP method.

**Figure
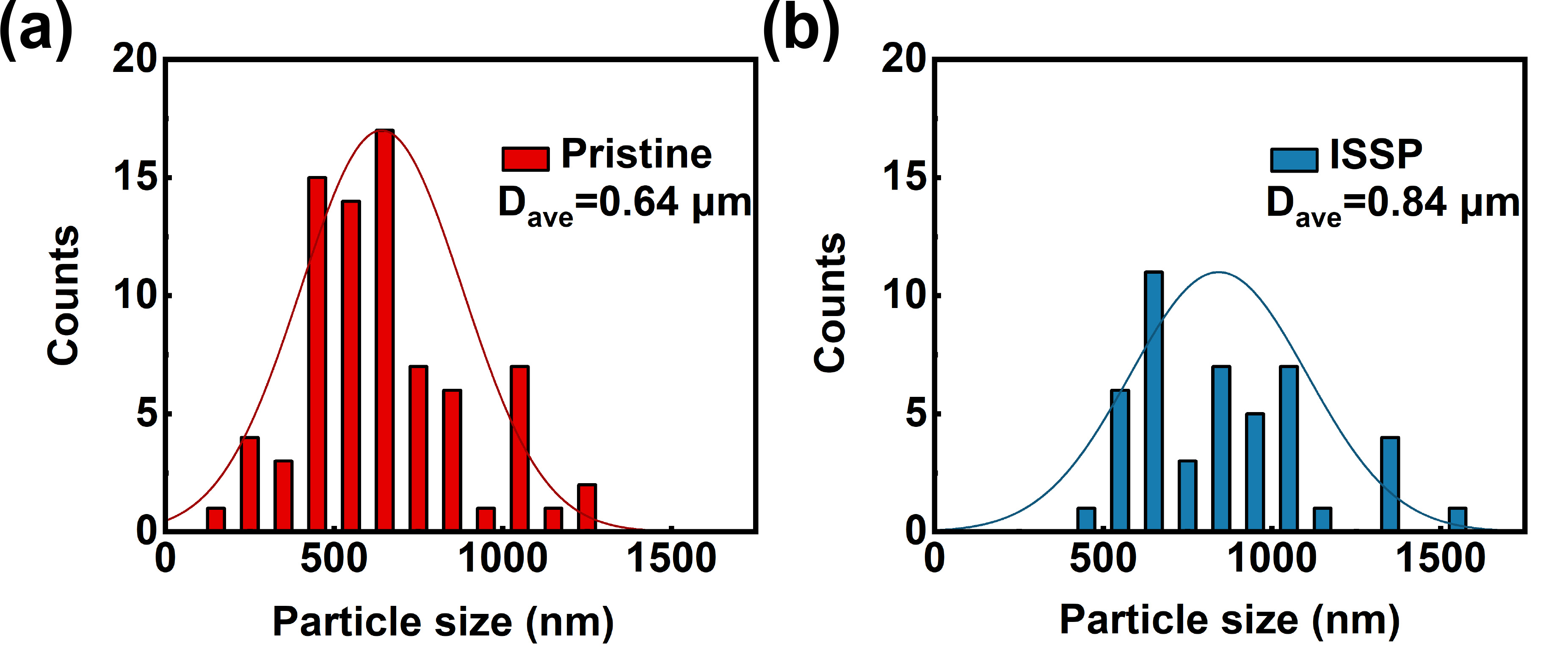
 S3.** Grain size measurements and analysis of a) the pristine and b) ISSP-treated perovskite films.

**
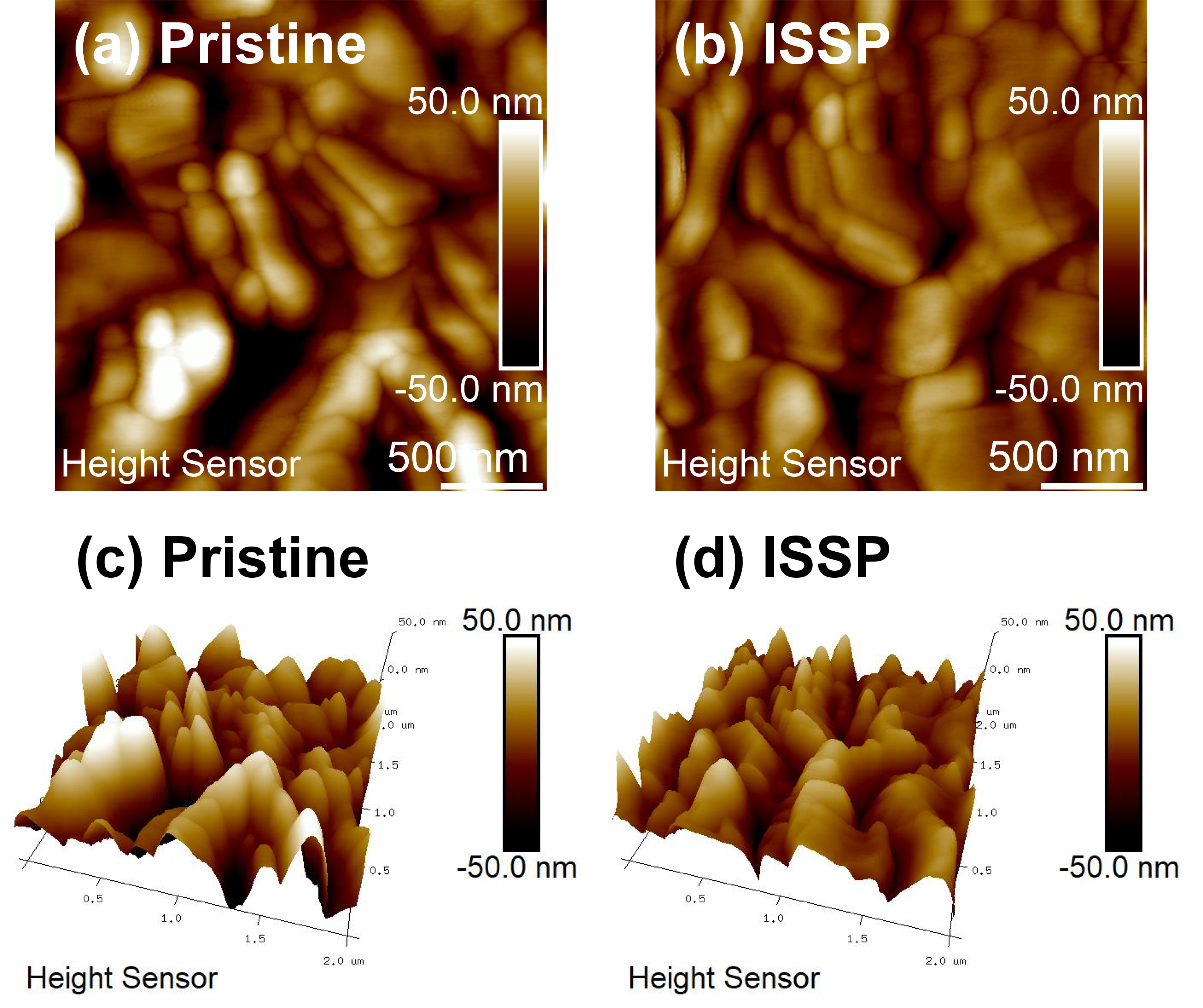
Figure S4.** 2D AFM images of a) the pristine and b) ISSP-treated perovskite films. 3D AFM images of c) the pristine and d) ISSP-treated perovskite films.


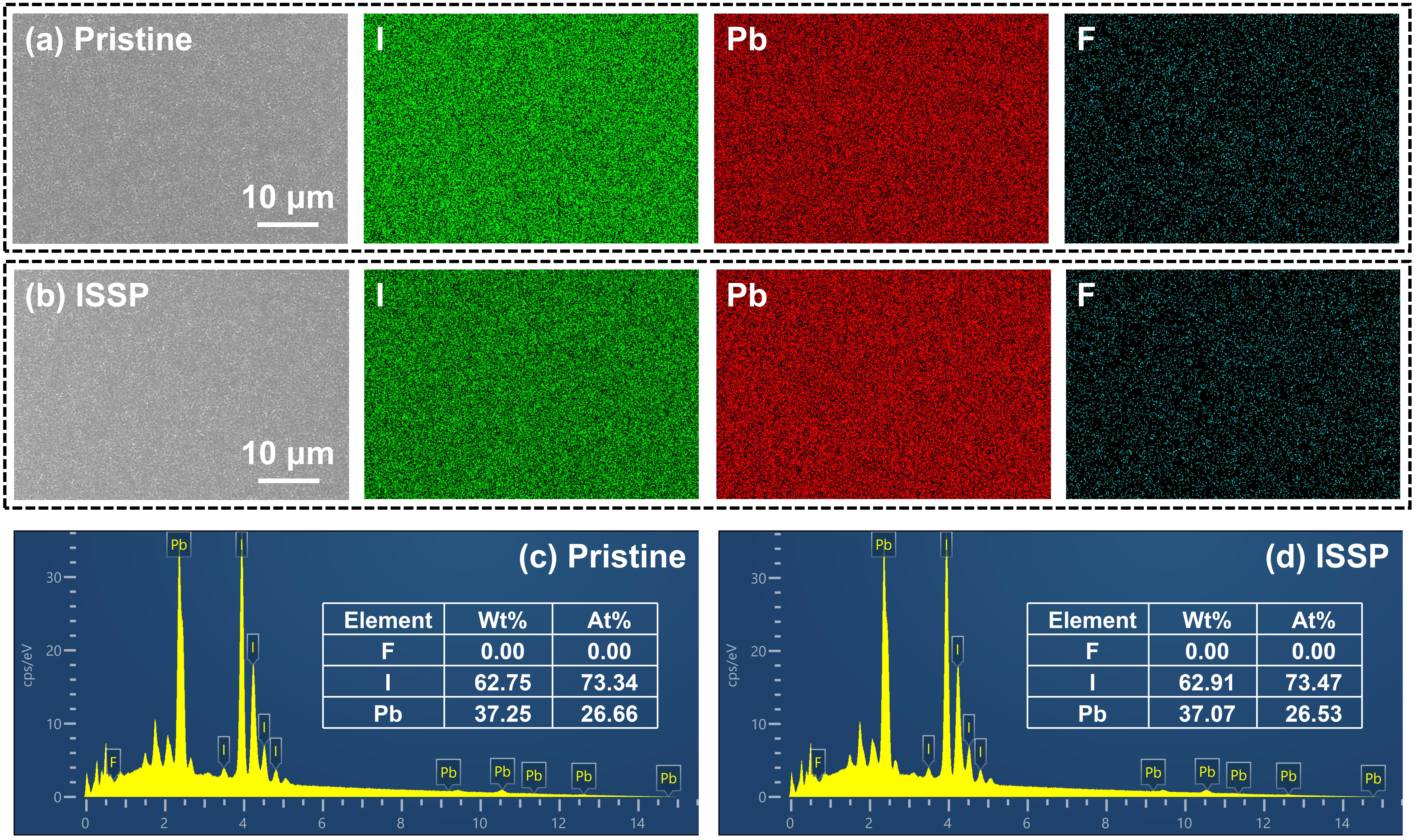


**Figure S5.** SEM-EDS mapping of I (green), Pb (red), and F (cyan)) elements of the a) pristine and b) ISSP-treated perovskite films. Elemental content analysis of the c) pristine and d) ISSP-treated perovskite films.

**
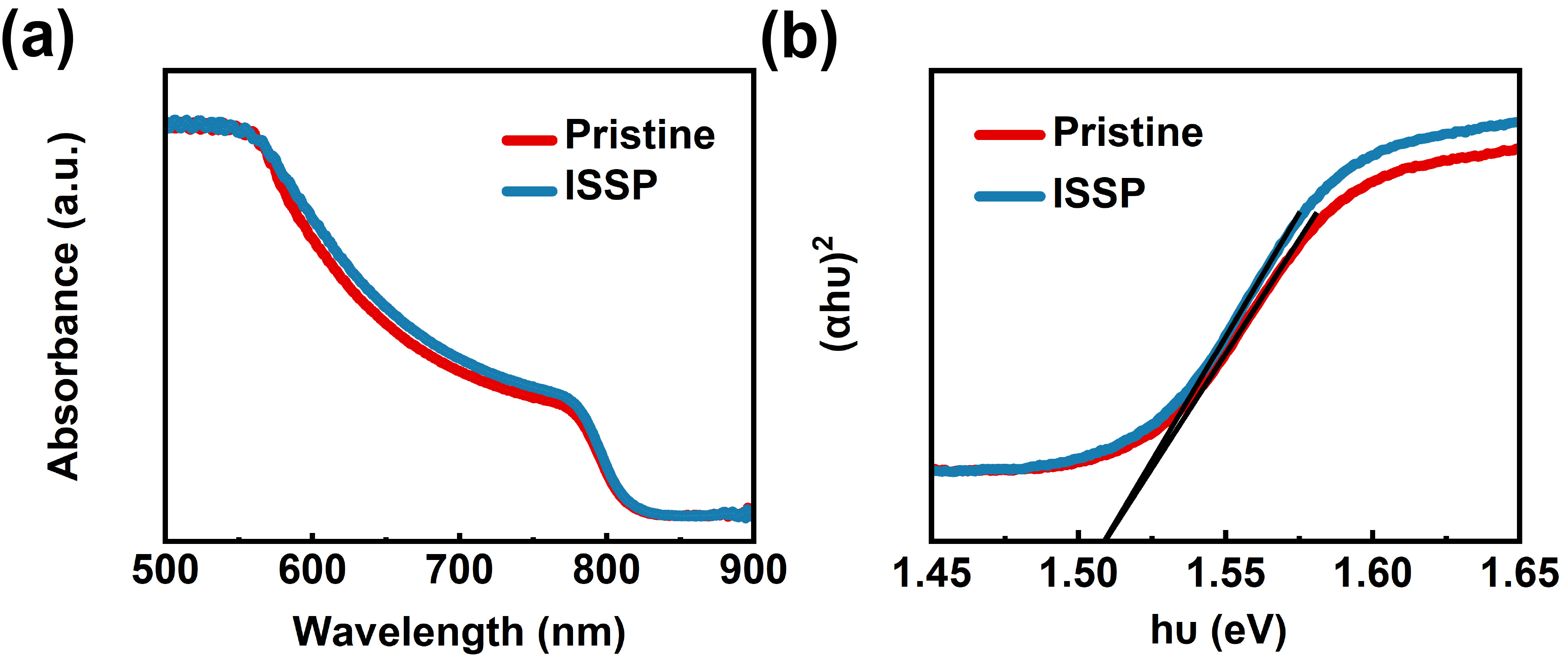
Figure S6.** a) UV-vis absorption spectra and b) the corresponding tauc plots of the pristine and ISSP-treated perovskite films.

**
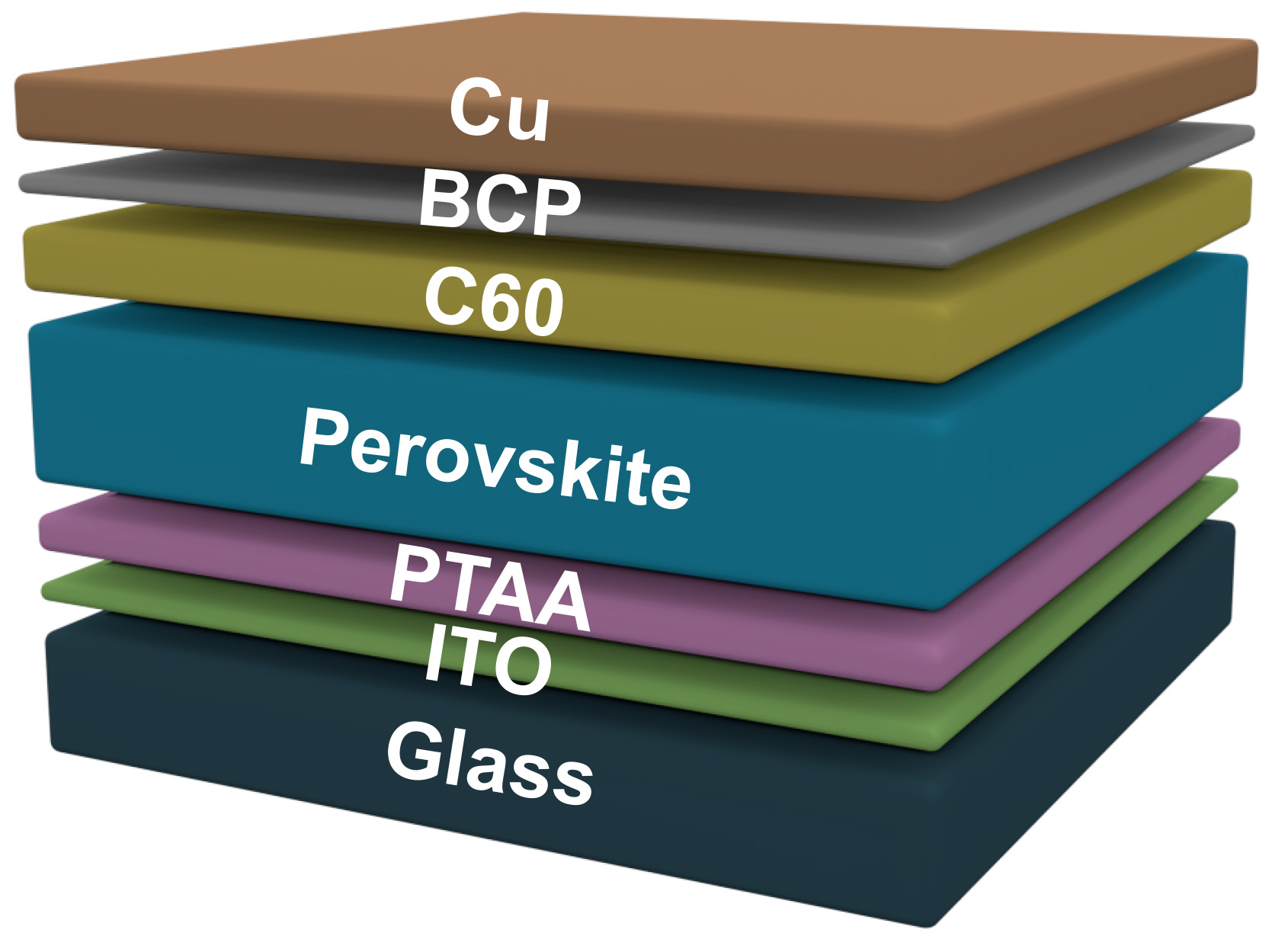
Figure S7.** Structure schematic of the PSCs.

**
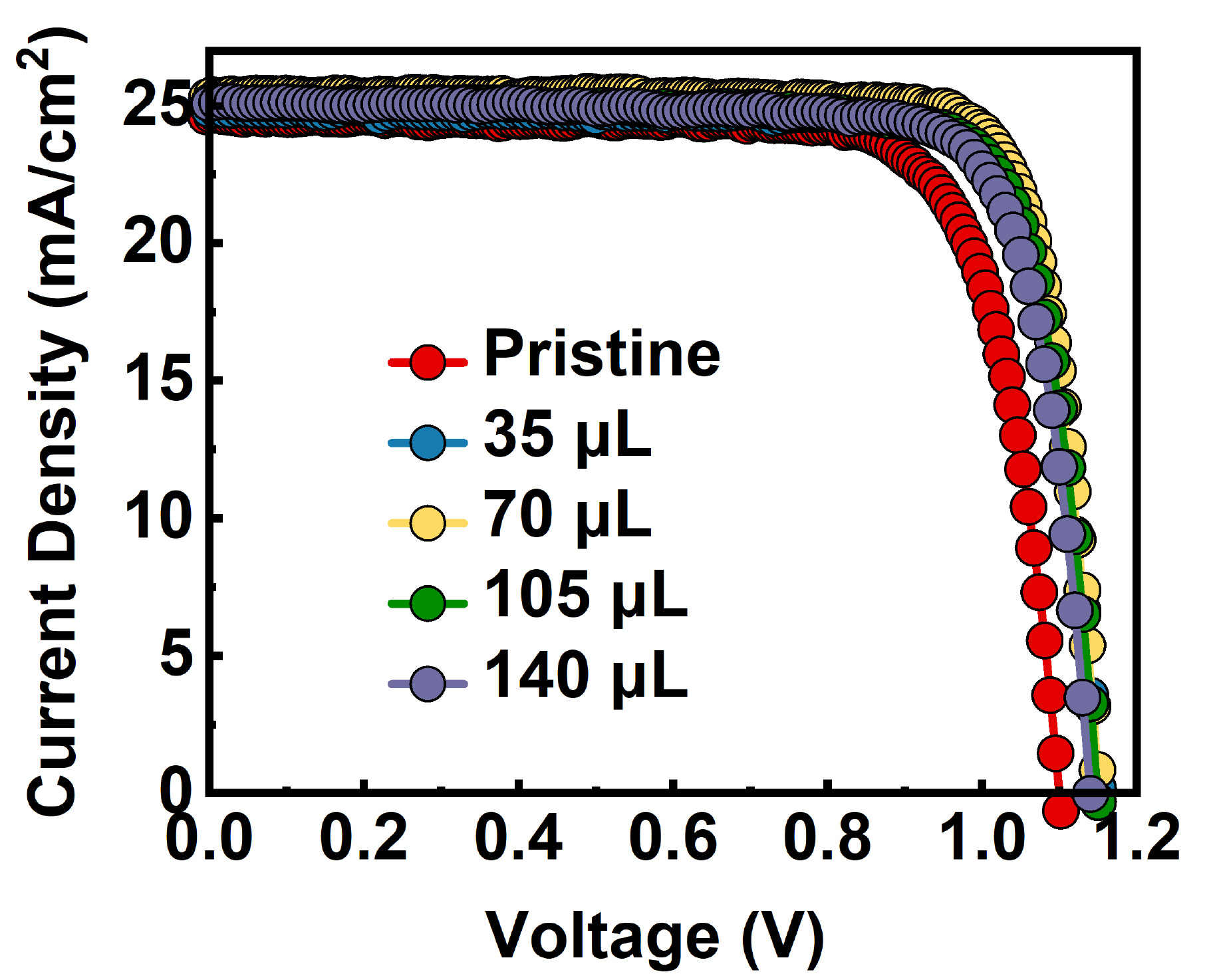
Figure S8.** The *J-V* curves of the pristine and ISSP-treated PSCs with various volumes of HFIP.

**
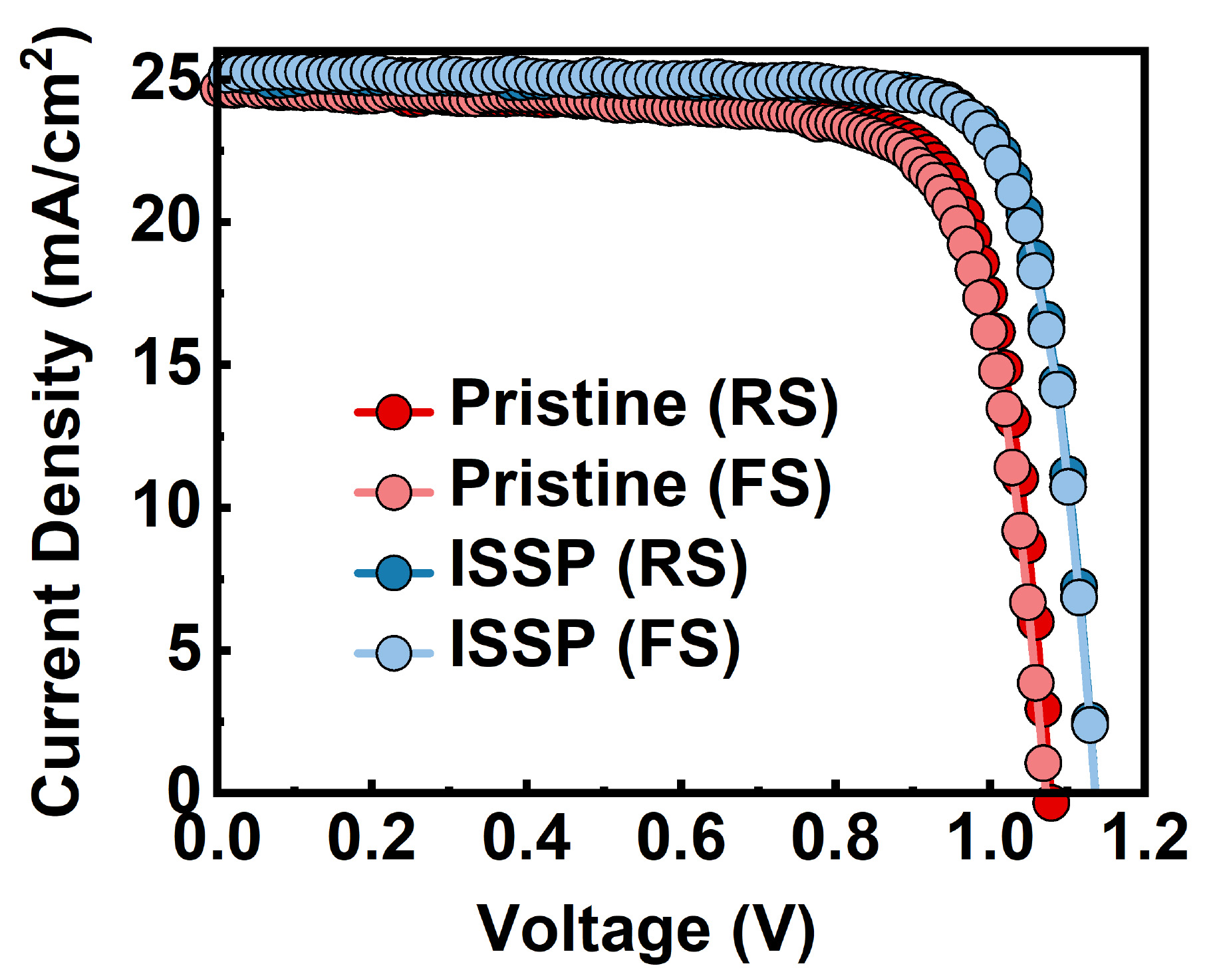
Figure S9.** The reverse and forward *J-V* curves of the pristine and ISSP-treated PSCs.

**
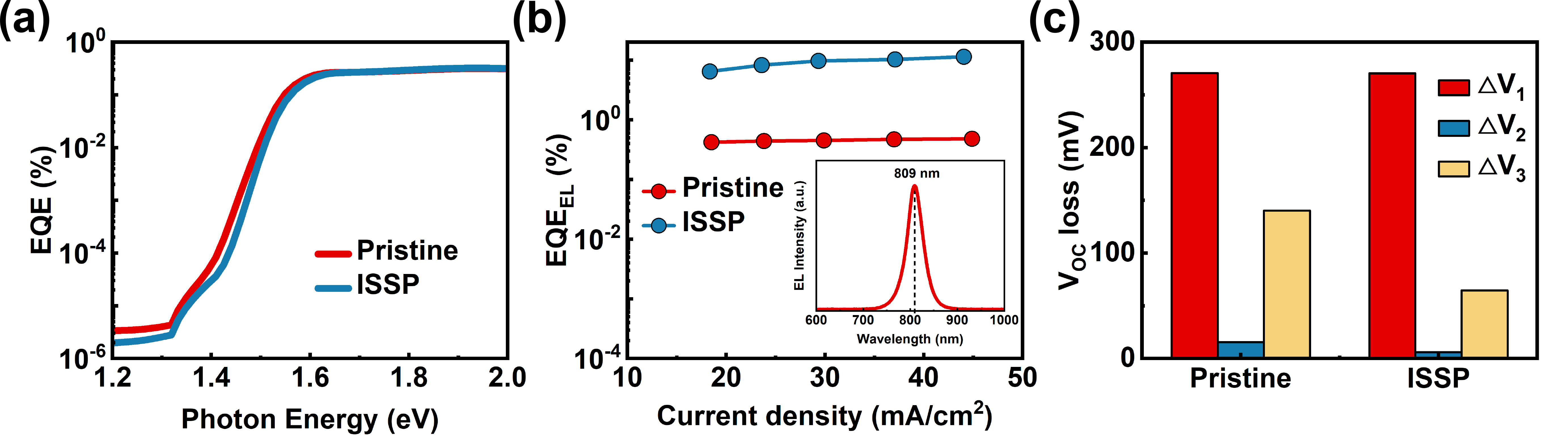
Figure S10.** a) Highly sensitive EQE spectra, b) EQE_EL_ and the EL spectra and c) V_OC_ loss analysis of the pristine and ISSP-treated devices.

**Figure
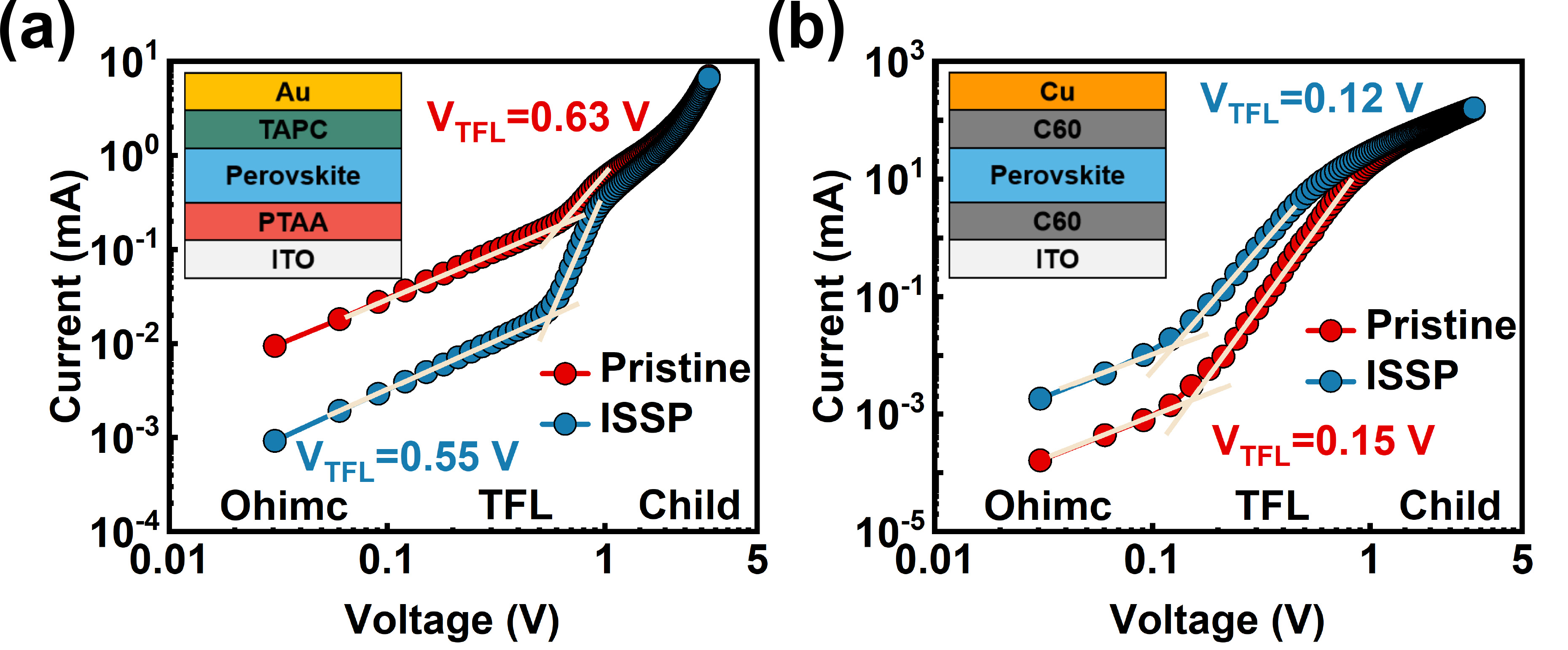
 S11.** SCLC curves for a) hole-only devices with the structure of ITO/PTAA/Perovskite/TAPC/Au and b) electron-only devices with the structure of ITO/C60/Perovskite/C60/Cu.

**Figure**
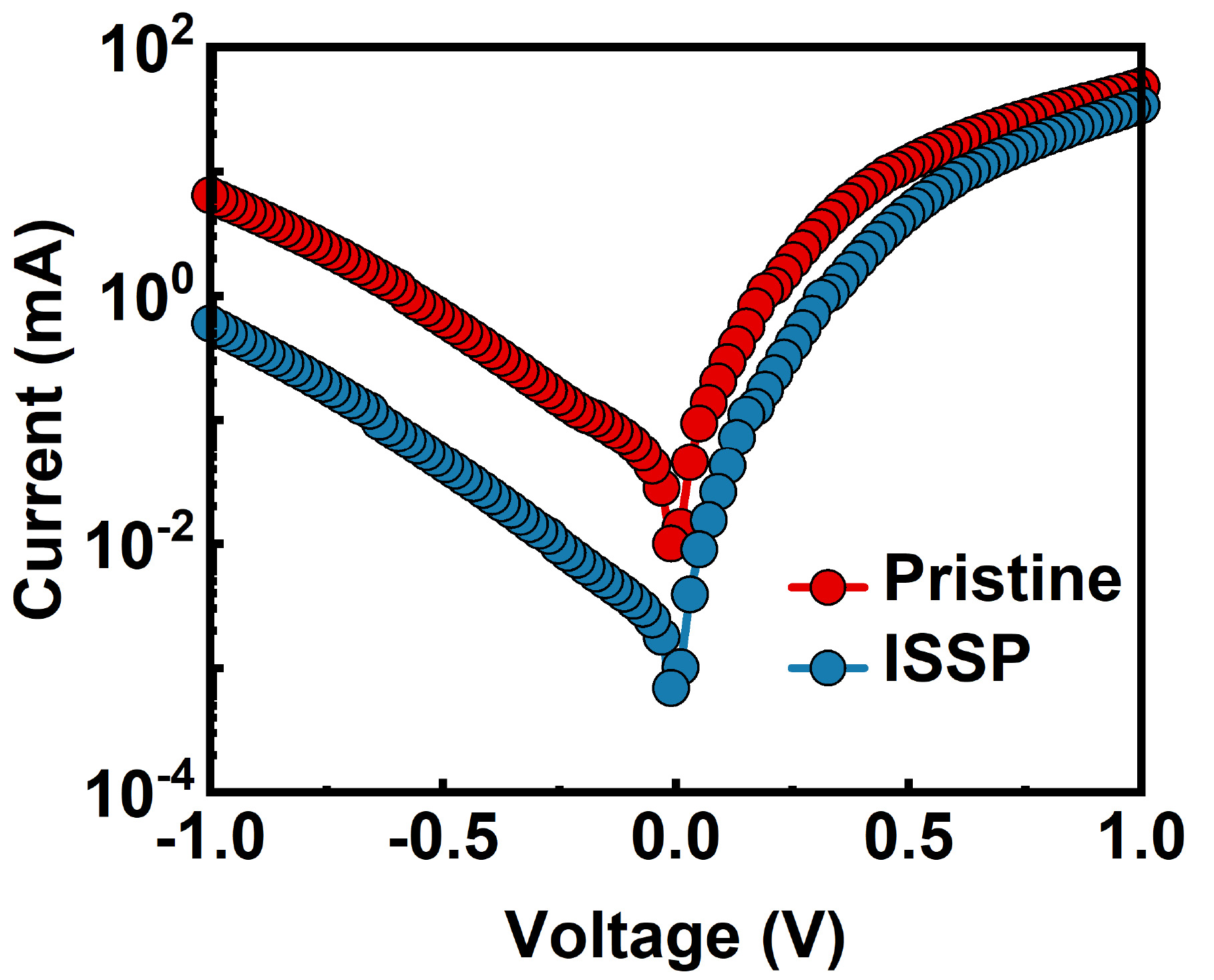
 **S12.** Dark *J-V* curves for the PSCs.

**Figure
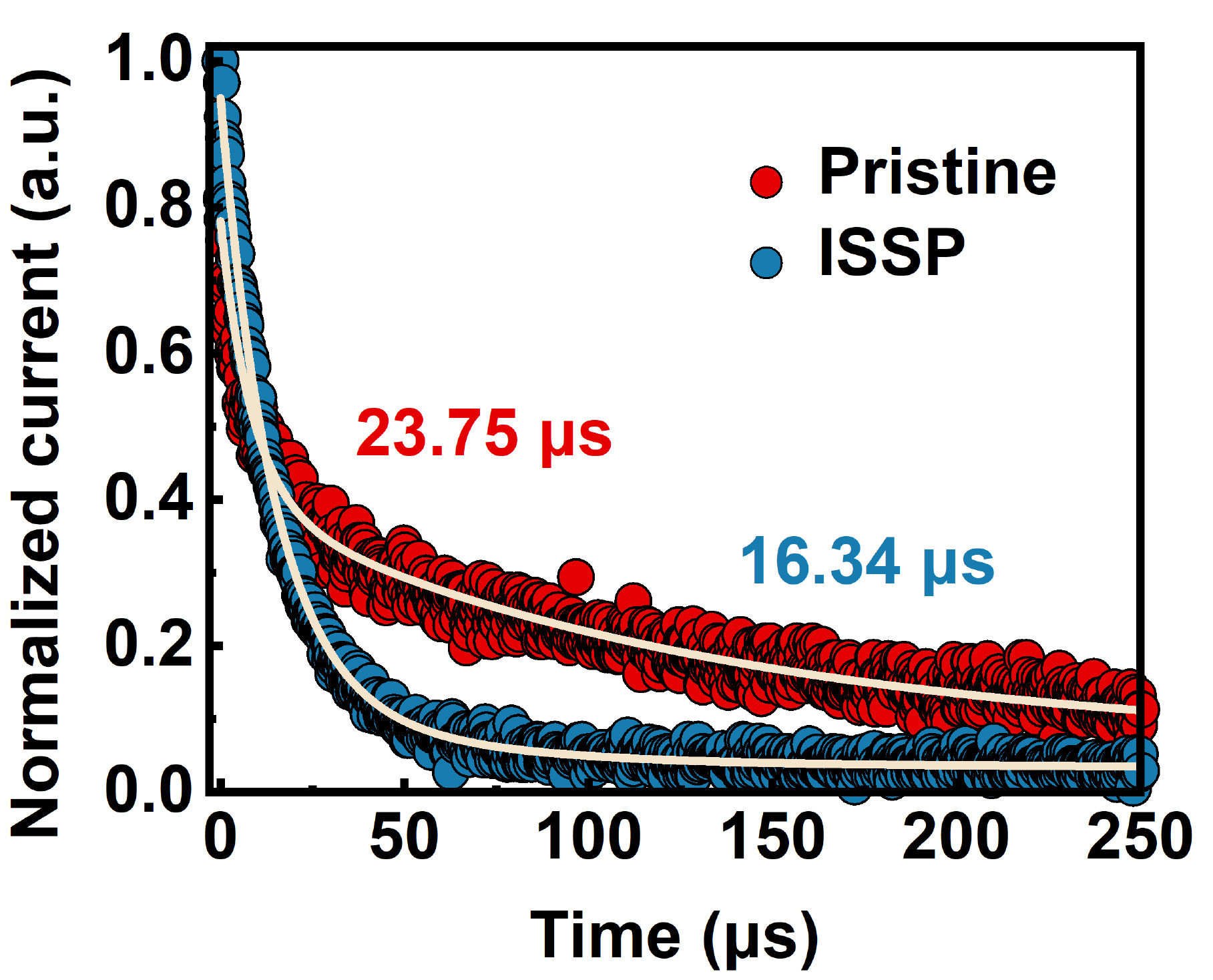
 S13.** TPC decay spectra of the PSCs.

**Figure
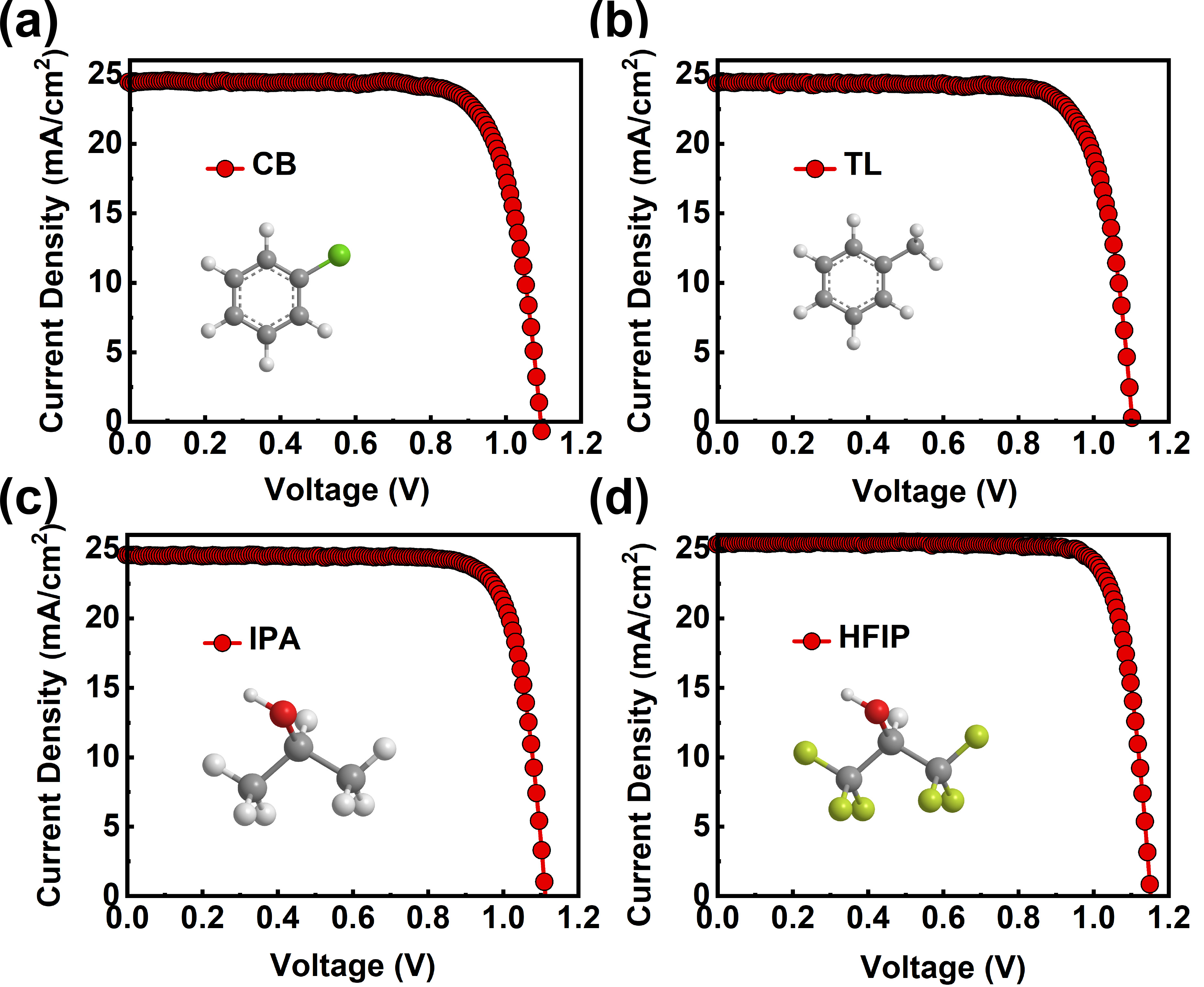
 S14.** The *J-V* curves of the PSCs dynamically spin-coated with different surface polishing agent.

**Figure
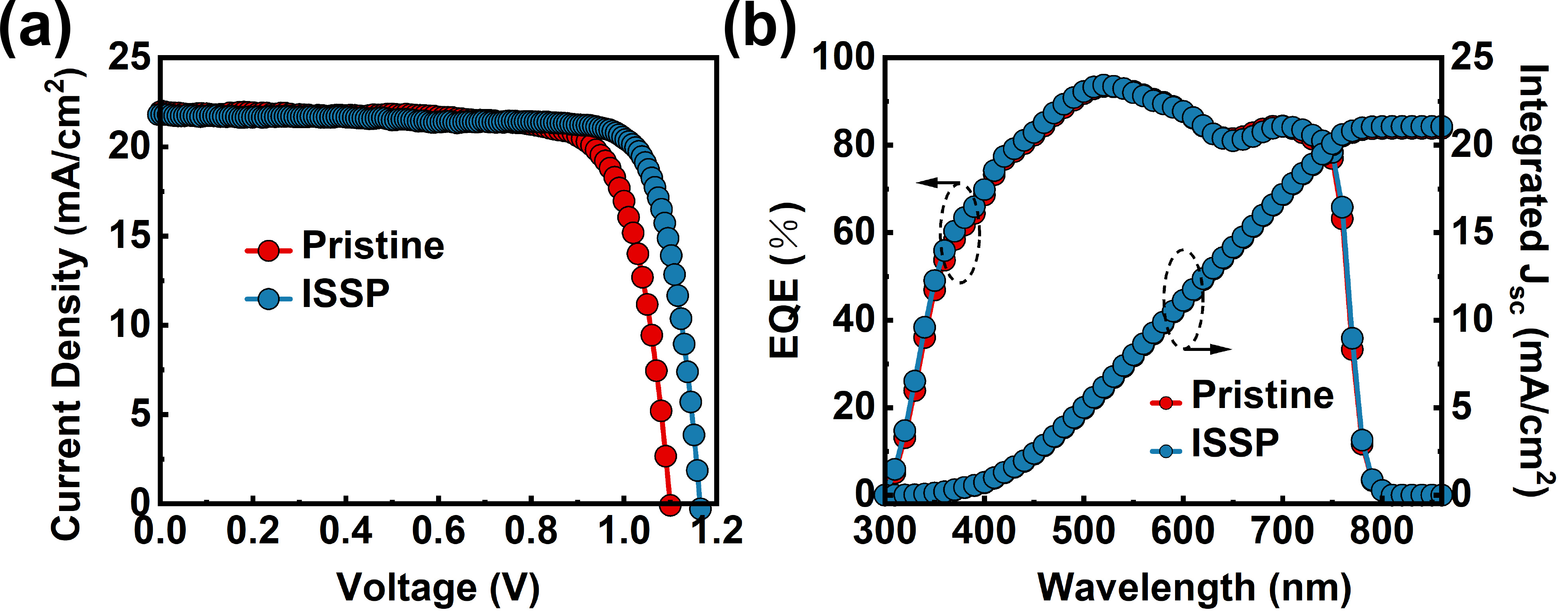
 S15.** a) The *J-V* curves and b) the corresponding EQE of Cs_0.05_(FA_0.83_MA_0.17_)_0.95_Pb(I_0.83_Br_0.17_)_3_ PSCs with and without ISSP treatment. The structure of the Cs_0.05_(FA_0.83_MA_0.17_)_0.95_Pb(I_0.83_Br_0.17_)_3_ PSCs is glass/ITO/PTAA/Perovskite/C60/BCP/Cu.

**Figure
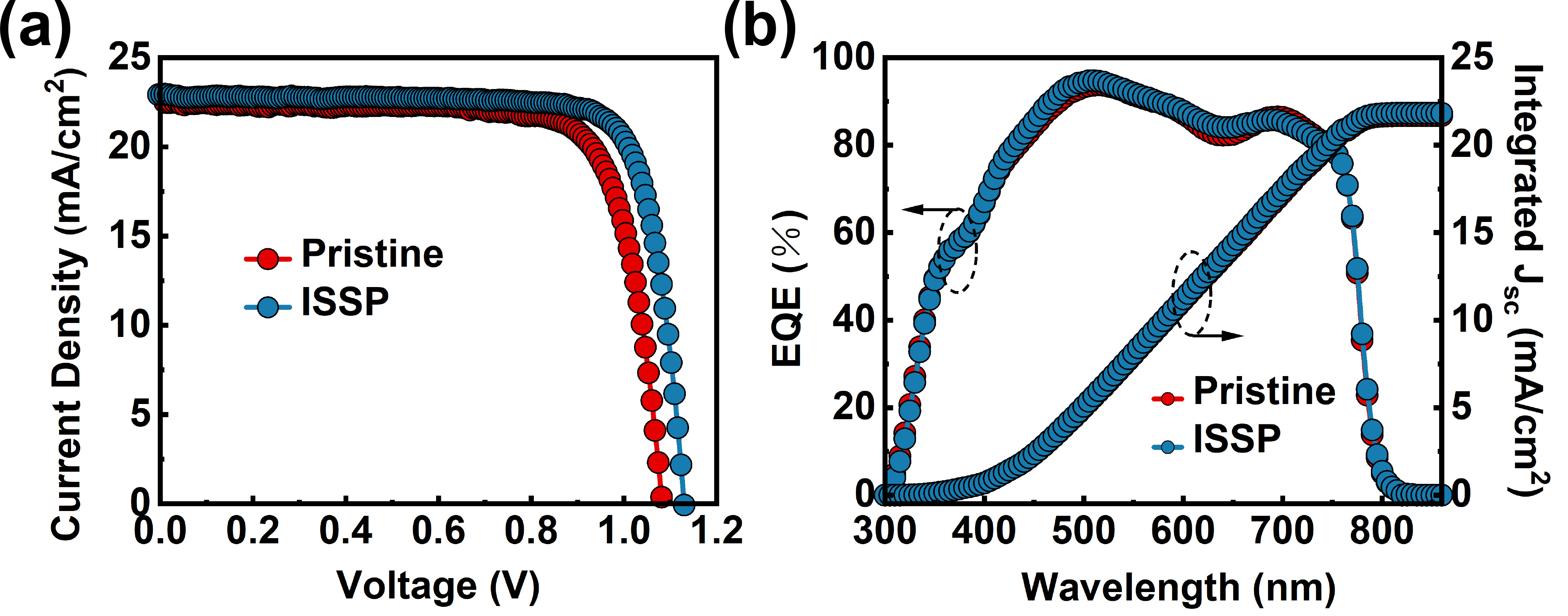
 S16.** a) The *J-V* curves and b) the corresponding EQE of MAPbI_3_ PSCs with and without ISSP treatment. The structure of the MAPbI_3_ PSCs is glass/ITO/PTAA/Perovskite/C60/BCP/Cu.


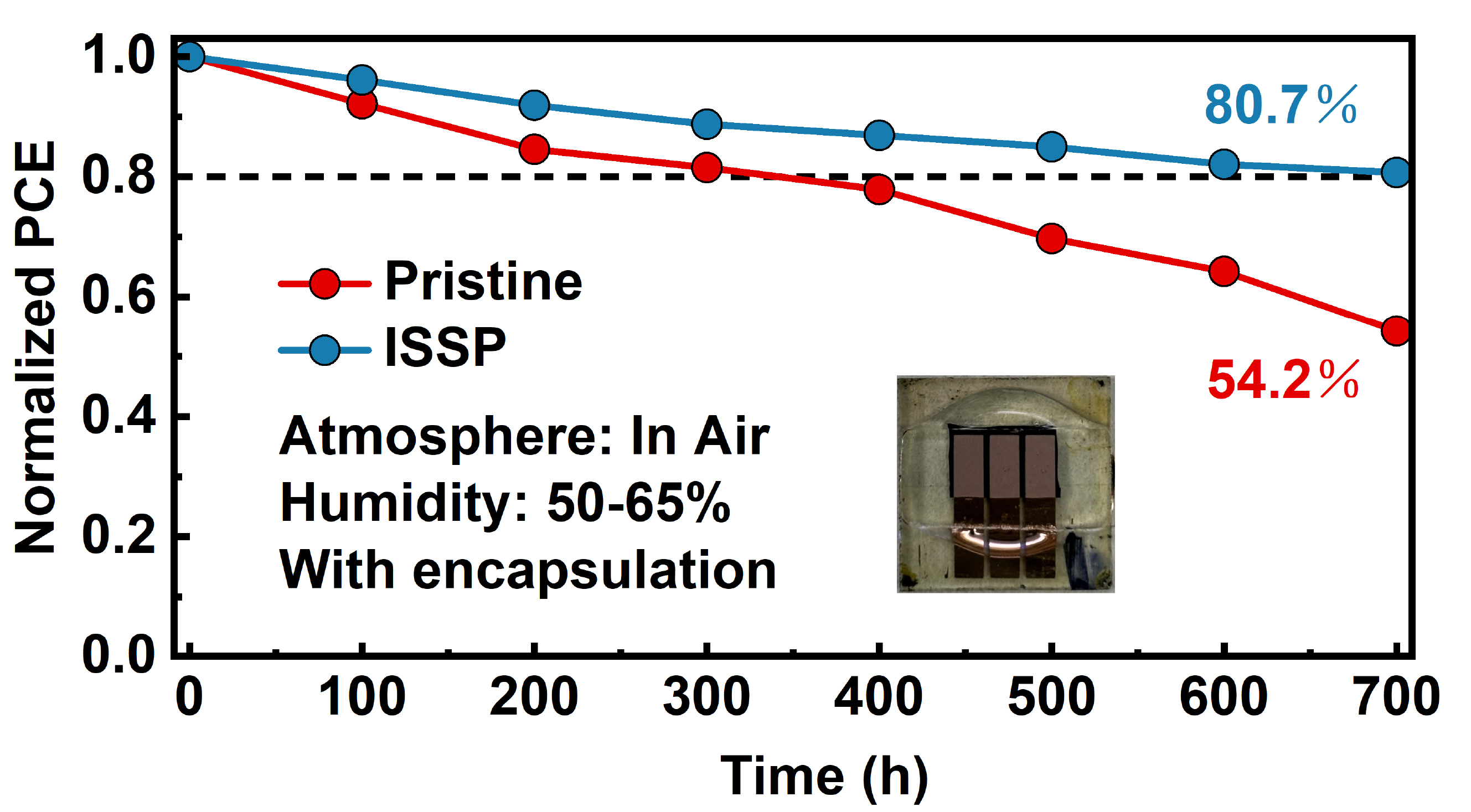
**Figure S17.** Normalized PCE of the encapsulated PSCs storage in humid air environment (RH of 50-65%).

**Figure
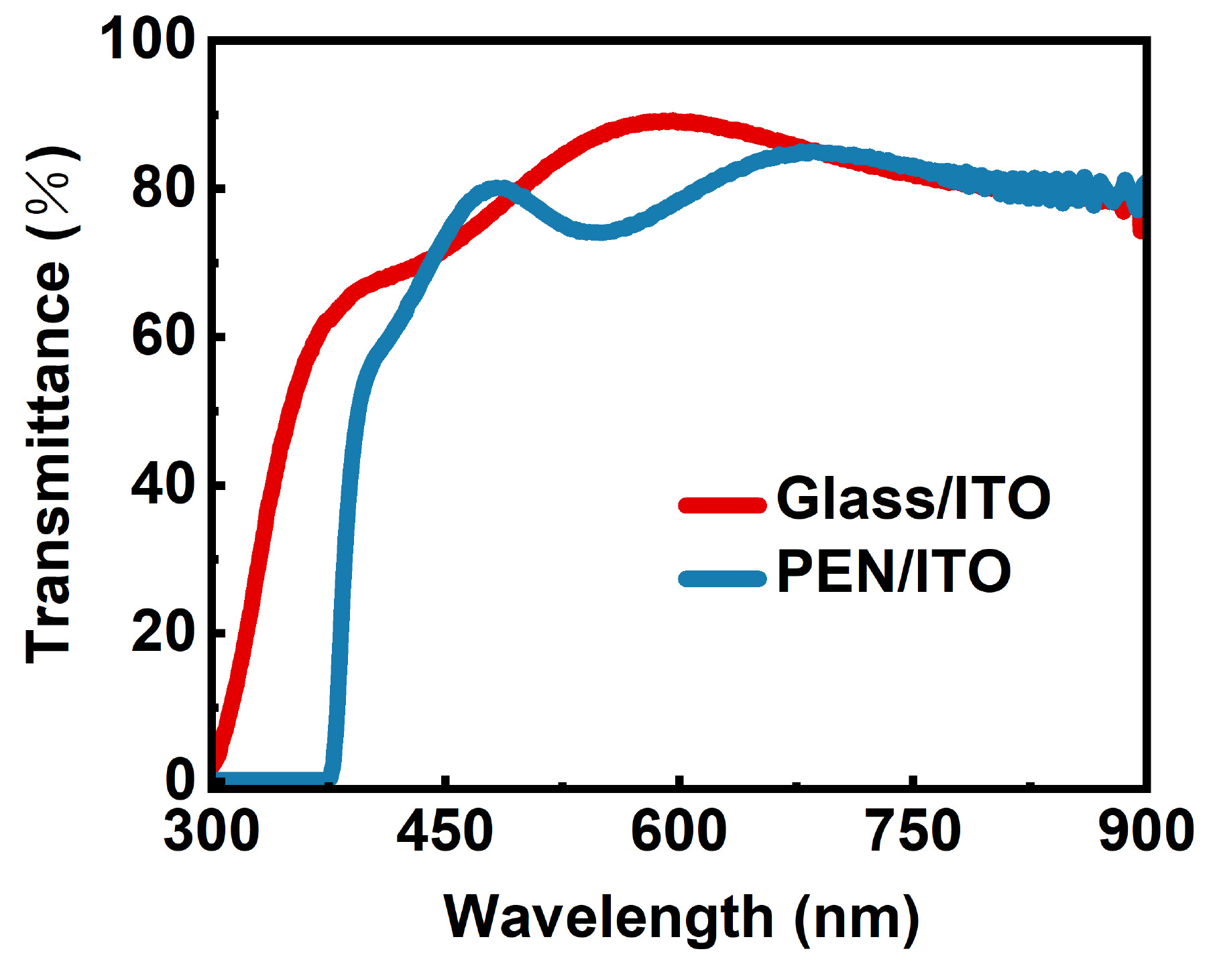
 S18.** The transmittance spectra of Glass/ITO and PEN/ITO.

**Supplementary Note 1**

The defect density (*N_trap_*) can be calculated using the turning point (*V_TFL_*) between the ohmic region and the trap-filled limit region (*TFL*) region based on the following equation:^[1]^

$\text{N}_{\text{trap}}\text{ }=\frac{\text{2εε}_{0}\text{V}_{\text{TFL}}}{\text{eL}^{2}}\text{ }$ (S1)

where *ε_0_* and *ε* refer to the vacuum permittivity and the relative dielectric constant of the perovskite, respectively, *e* is the elementary charge, and *L* refers to the thickness of the perovskite film. The ISSP-treated hole-only devices exhibit a decreased *N_trap_* from 9.36×10^15^ cm^-3^ to 8.81×10^15^ cm^-3^. Meanwhile, the electron-only devices show a similar trend, with the corresponding *N_trap_* are 2.23×10^15^ cm^-3^ and 1.92×10^15^ cm^-3^ without and with ISSP treatment, respectively.

**Supplementary Note 2:**

The detailed balance theory is utilized to calculate the *V_OC_* loss:^[2]^

$$q\Delta V=E_{g}-V_{\mathrm{OC}}$$

$$=\left( E_{g}-qV_{\mathrm{OC}}^{\mathrm{SC}} \right)+\left( qV_{\mathrm{OC}}^{\mathrm{SC}}-qV_{\mathrm{OC}}^{\mathrm{rad}} \right)+(qV_{\mathrm{OC}}^{\mathrm{rad}}-qV_{\mathrm{OC}})$$

$$=\left( E_{g}-qV_{\mathrm{OC}}^{\mathrm{SC}}+q\Delta V_{\mathrm{OC}}^{\mathrm{SC}} \right)+q\Delta V_{\mathrm{OC}}^{\mathrm{rad}}+q\Delta V_{\mathrm{OC}}^{non-rad}$$

$=q(\Delta V_{1}+\Delta V_{2}+\Delta V_{3})$ (S2)

where q is an elementary charge and E_g_ denotes the perovskite bandgap. $\Delta V$ denotes the total voltage loss, $V_{\mathrm{OC}}^{\mathrm{SC}}$ denotes the Shockley-Queisser voltage limit, $V_{\mathrm{OC}}^{\mathrm{rad}}$ denotes the *V_OC_* in the radiative limit, $\Delta V_{\mathrm{OC}}^{\mathrm{SC}}$ denotes the voltage loss associated with the non-ideal EQE above the bandgap, $\Delta V_{\mathrm{OC}}^{\mathrm{rad}}$ denotes the voltage loss due to radiative recombination in the sub-bandgap region, $\Delta V_{\mathrm{OC}}^{non-rad}$ denotes the voltage loss caused by nonradiative recombination.

$\Delta V_{1}$ issues from the inevitable radiative recombination and the non-ideal EQE above E_g_. The pristine and ISSP-treated PSCs exhibit approximate $\Delta V_{1}$ of 0.271 V and 0.270 V, respectively. Energy loss coming from black-body radiation causes $\Delta V_{2}$ to be negative, in which case the EQE of devices stretch into the area beneath E_g_.^[3, 4]^ The values of $\Delta V_{2}$ are 0.016 V and 0.006 V, respectively, which is determined by the highly sensitive EQE (Figure 10a). The tiny $\Delta V_{2}$ value measured in ISSP-treated PSCs can be attributed to the sheer absorption edge of perovskite absorber in agreement with the prior research.^[5-7]^ $\Delta V_{3}$ is the voltage loss resulting from non-recombination, and it can be calculated using the equation:^[8]^

$\Delta V_{3}=-\frac{k_{B}T}{q}\ln\left( \mathrm{EQE}_{\mathrm{EL}} \right)$ （S3）

Here, k_B_ denotes the Boltzmann constant and the value of EQE_EL_ (EQE of electroluminescence) was calculated by employing photovoltaic solar cells as light-emitting diodes (LEDs).^[9]^ As shown in Figure S10b, the EQE_EL_ of the ISSP-treated PSCs is 8.22% with the EL spectrum peaking about 809 nm, which is equivalent to a $\Delta V_{3}$ value of 0.64 V, while the EQE_EL_ of the pristine PSCs is almost declined to 0.44% with a value of 0.14 V. Their parameters of *V_OC_* loss are revealed in Figure S10c and summarized in **Table S7**. Therefore, the $\Delta V_{3}$ is responsible for most of the voltage loss of device and a significantly decreased *V_OC_* loss of 0.35 V is achievable in ISSP-treated PSCs, demonstrating the outstanding passivating impact of ISSP treatment in minimizing non-radiative recombination loss.

According to the reciprocity relationship between photovoltaic external quantum efficiency ($\mathrm{EQE}_{\mathrm{PV}}$) and electroluminescence (EL), the *V*_OC_ of a solar cell can be calculated with the equation listed below:^[10, 11]^

$V_{\mathrm{OC}}=\frac{k_{B}T}{q}\ln\left( \frac{J_{\mathrm{SC}}}{J_{0}} \right)$ (S4)

where $q$ is element charge, $k_{B}$ is Boltzmann constant, $T$ is temperature, *J*_SC_ is short-circuit current, *J*_0_ is dark saturation current. The expressions of *J*_SC_ and *J*_0_ are given by:

$J_{\mathrm{SC}}=q\int_{0}^{\infty} \mathrm{EQE}_{\mathrm{PV}}\left( E \right)\phi_{AM1.5}\left( E \right)dE$ (S5)

$J_{0}=\frac{q}{{EQE}_{\mathrm{EL}}}\int_{0}^{\infty} \mathrm{EQE}_{\mathrm{PV}}\left( E \right)\phi_{\mathrm{BB}}\left( E \right)dE$ (S6)

$\phi_{\mathrm{BB}}\left( E \right)=\frac{2\pi E^{2}}{h^{3}c^{2}}\frac{1}{\exp\left( \frac{E}{k_{B}T} \right)-1}$ (S7)

where $\mathrm{EQE}_{\mathrm{EL}}$ is EL external quantum efficiency, $\phi_{AM1.5}$ is solar cell radiative spectrum, $\phi_{\mathrm{BB}}$ is black-body radiative spectrum, $c$ is light speed in vacuum.

In Schokley-Queisser limit (S-Q limit): (1) The $\mathrm{EQE}_{\mathrm{PV}}$ is described with Heaviside step function, where $\mathrm{EQE}_{\mathrm{PV}}\left( E \right)=\left\{ \begin{aligned} 1, E\geq E_{g} \\ 0, E<E_{g} \end{aligned} \right.$; (2) only the photos with energy larger than bandgap ($E_{g}$) are absorbed; (3) all recombination is radiative ($\mathrm{EQE}_{\mathrm{EL}}=1$). Therefore, *J*_SC_ and *J*_0_ in S-Q limit are written as:

$J_{\mathrm{SC}}^{\mathrm{SQ}}=q\int_{E_{g}}^{\infty} \phi_{AM1.5}\left( E \right)dE$ (S8)

$J_{0}^{\mathrm{SQ}}=q\int_{E_{g}}^{\infty} \phi_{\mathrm{BB}}\left( E \right)dE$ (S9)

Therefore, *V*_OC_ in S-Q limit is:

$V_{\mathrm{OC}}^{\mathrm{SQ}}=\frac{k_{B}T}{q}\ln\left( \frac{J_{\mathrm{SC}}^{\mathrm{SQ}}}{J_{0}^{\mathrm{SQ}}} \right)$ (S10)

Considering the assumption of S-Q limit, $V_{\mathrm{OC}}^{\mathrm{SQ}}$ can be degraded to *V*_OC_ with 3 loss components. The first *V*_OC_ loss component, ${\Delta V}_{\mathrm{OC}}^{\mathrm{SC}}$, is due to the non-ideal ${EQE}_{\mathrm{PV}}$, which is less than 100%. In this condition, short-circuit current is expressed as:

$J_{\mathrm{SC}}=q\int_{0}^{\infty} \mathrm{EQE}_{\mathrm{PV}}\left( E \right)\phi_{AM1.5}\left( E \right)dE$ (S11)

${\Delta V}_{\mathrm{OC}}^{\mathrm{SC}}$ is calculated as:

${\Delta V}_{\mathrm{OC}}^{\mathrm{SC}}=V_{\mathrm{OC}}^{\mathrm{SQ}}-\frac{k_{B}T}{q}\ln\left( \frac{J_{\mathrm{SC}}}{J_{0}^{\mathrm{SQ}}} \right)=\frac{k_{B}T}{q}\ln\left( \frac{J_{\mathrm{SC}}^{\mathrm{SQ}}}{J_{\mathrm{SC}}} \right)$ (S12)

The second *V*_OC_ loss component comes from the energy loss associated with extra thermal radiation of solar cell in dark. In experiment, the $\mathrm{EQE}_{\mathrm{PV}}$ extends into the sub-bandgap region, where the black-body radiation increases with the photo energy lowering. Thus, this sub-bandgap $\mathrm{EQE}_{\mathrm{PV}}$ increased the dark saturation current. The dark saturation current in this condition is written as:

$J_{0}^{\mathrm{rad}}=q\int_{0}^{\infty} \mathrm{EQE}_{\mathrm{PV}}\left( E \right)\phi_{\mathrm{BB}}\left( E \right)dE$ (S13)

therefore, the radiative *V*_OC_ loss, ${\Delta V}_{\mathrm{OC}}^{\mathrm{rad}}$, is:

${\Delta V}_{\mathrm{OC}}^{\mathrm{rad}}=\frac{k_{B}T}{q}\ln\left( \frac{J_{\mathrm{SC}}}{J_{0}^{\mathrm{SQ}}} \right)-\frac{k_{B}T}{q}\ln\left( \frac{J_{\mathrm{SC}}}{J_{0}^{\mathrm{rad}}} \right)=\frac{k_{B}T}{q}\ln\left( \frac{J_{0}^{\mathrm{rad}}}{J_{0}^{\mathrm{SQ}}} \right)$ (S14)

The third *V*_OC_ loss component,${\Delta V}_{\mathrm{OC}}^{\mathrm{nonrad}}$, is ascribed to the non-radiative recombination in device, which can be calculated as:

${\Delta V}_{\mathrm{OC}}^{\mathrm{nonrad}}=\frac{k_{B}T}{q}\ln\left( \frac{J_{\mathrm{SC}}}{J_{0}^{\mathrm{rad}}} \right)-V_{\mathrm{OC}}$ (S15)

According to Equation 4 and 10, $J_{0}^{\mathrm{rad}}={\mathrm{EQE}_{\mathrm{EL}}\cdot J}_{0}$, so combining Equation 2, Equation 12 can be rewritten as:

${\Delta V}_{\mathrm{OC}}^{\mathrm{nonrad}}=\frac{k_{B}T}{q}\ln\left( \frac{J_{\mathrm{SC}}}{{\mathrm{EQE}_{\mathrm{EL}}\cdot J}_{0}} \right)-\frac{k_{B}T}{q}\ln\left( \frac{J_{\mathrm{SC}}}{J_{0}} \right)=-\frac{k_{B}T}{q}\ln\left( \mathrm{EQE}_{\mathrm{EL}} \right)$ (S16)

**Table S1.** Electronic energy levels of perovskite films obtained from UPS.

| Sample | E_cutoff_ [eV] | E_onset_ [eV] | E_V_ [eV] | E_C_ [eV] | W_F_ [eV] |
| --- | --- | --- | --- | --- | --- |
| Pristine | 16.70 | 1.12 | -5.64 | -4.13 | -4.52 |
| ISSP-treated | 16.85 | 1.29 | -5.66 | -4.15 | -4.37 |

**Table S2.** The *J-V* parameters of PSCs with various volumes of ISSP treatment.

| Sample | *V_OC_* [V] | *J_SC_* [mA/cm^2^] | *FF* [%] | PCE [%] |
| --- | --- | --- | --- | --- |
| 0 μL | 1.10 | 24.63 | 77.06 | 20.88 |
| 35 μL | 1.15 | 24.85 | 81.16 | 23.21 |
| 70 μL | 1.15 | 25.30 | 82.82 | 24.13 |
| 105 μL | 1.15 | 25.14 | 80.63 | 23.29 |
| 140 μL | 1.14 | 25.07 | 80.42 | 22.99 |

**Table S3.** Summary of the performance of PSCs with polishing treatment.

| Device structure | *V_OC_* [V] | *J_SC_* [mA/cm^2^] | *FF* [%[ | PCE [%] | Ref |
| --- | --- | --- | --- | --- | --- |
| ITO/NiO_X_/CsPbI_2.85_Br_0.15_/PCBM/BCP/Cu | 1.196 | 19.30 | 83.79 | 19.34 | [12] |
| ITO/P3CT-N/CsPbI_3_/PCBM/C60/BCP/Ag | 1.213 | 20.44 | 80.37 | 19.84 | [13] |
| ITO/PTAA/BA_2_MA_4_Pb_5_I_16_/C60/BCP/Ag | 1.21 | 19.99 | 75.68 | 18.38 | [14] |
| ITO/PTAA/FA_0.85_Cs_0.15_PbI_3_/PCBM/Ag | 1.17 | 24.84 | 83.49 | 24.27 | [15] |
| ITO/PTAA/Cs_0.06_FA_0.79_MA_0.15_Pb(I_0.85_Br_0.15_)_3_/PCBM/BCP/Ag | 1.14 | 23.31 | 70.22 | 18.6 | [16] |
| ITO/PTAA/Rb_0.05_Cs_0.05_FA_0.85_MA_0.05_PbI_2.85_Br_0.15_/C60/BCP/Cu | 1.15 | 23.4 | 81.7 | 22.0 | [17] |
| FTO/SnO_2_/(FA_0.95_MA_0.05_PbI_2.85_Br_0.15_/Spiro-OMeTAD/Au | 1.14 | 25.27 | 82.01 | 23.58 | [18] |
| FTO/c-TiO_2_/m-TiO_2_/FAPbI_3_/Spiro-OMeTAD/Au | 1.15 | 25.86 | 82.37 | 24.50 | [19] |
| ITO/SnO_2_/Cs_0.05_(MA_0.17_FA_0.83_)_0.95_Pb(I_0.83_Br_0.17_)_3_/Spiro-OMeTAD/Au | 1.19 | 21.0 | 77.30 | 19.30 | [20] |
| ITO/PTAA/MAPbI_3_/PCBM/TiO_X_/Cu | 1.12 | 21.64 | 77.9 | 18.89 | [21] |
| ITO/PTAA/MAPbI_3_/PCBM/C60/BCP/Cu | 1.11 | 22.5 | 81.6 | 20.4 | [22] |
| ITO/PTAA/FA_0.92_MA_0.08_PbI_3_/C60/BCP/Cu | 1.15 | 25.30 | 82.82 | 24.13 | This Work |

**Table S4.** The reverse and forward *J-V* parameters of the pristine and ISSP-treated PSCs.

| Sample | Scan direction | *V_OC_* [V] | *J_SC_* [mA/cm^2^] | *FF* [%] | PCE [%] | HI [%] |
| --- | --- | --- | --- | --- | --- | --- |
| Pristine | Reverse | 1.08 | 24.67 | 77.41 | 20.59 | 2.72 |
|  | Forward | 1.07 | 24.68 | 75.65 | 20.03 |  |
| ISSP-treated | Reverse | 1.14 | 25.17 | 80.98 | 23.17 | 0.52 |
|  | Forward | 1.14 | 25.17 | 80.53 | 23.05 |  |

**Table S5.** The *J-V* parameters of the 20 individual pristine PSCs.

| Sample | *V_OC_* [V] | *J_SC_* [mA/cm^2^] | *FF* [%] | PCE [%] |
| --- | --- | --- | --- | --- |
| 1 | 1.10 | 24.63 | 77.06 | 20.88 |
| 2 | 1.08 | 24.02 | 77.11 | 19.93 |
| 3 | 1.09 | 24.38 | 77.71 | 20.66 |
| 4 | 1.08 | 24.09 | 76.13 | 19.85 |
| 5 | 1.07 | 24.51 | 76.98 | 20.24 |
| 6 | 1.08 | 24.39 | 77.31 | 20.36 |
| 7 | 1.08 | 24.09 | 74.90 | 19.50 |
| 8 | 1.08 | 24.28 | 78.07 | 20.42 |
| 9 | 1.09 | 24.74 | 76.31 | 20.53 |
| 10 | 1.08 | 24.67 | 77.41 | 20.59 |
| 11 | 1.07 | 24.54 | 77.01 | 20.31 |
| 12 | 1.06 | 24.12 | 76.30 | 19.54 |
| 13 | 1.07 | 24.58 | 77.26 | 20.23 |
| 14 | 1.09 | 24.37 | 78.22 | 20.80 |
| 15 | 1.10 | 24.12 | 75.05 | 19.92 |
| 16 | 1.10 | 24.56 | 75.45 | 20.37 |
| 17 | 1.10 | 23.93 | 76.13 | 20.07 |
| 18 | 1.09 | 24.27 | 76.94 | 20.35 |
| 19 | 1.08 | 24.11 | 78.24 | 20.43 |
| 20 | 1.07 | 23.84 | 76.90 | 19.63 |
| Average | 1.08 | 24.31 | 76.83 | 20.23 |

**Table S6.** The *J-V* parameters of the 20 individual ISSP-treated PSCs.

| Sample | *V_OC_* [V] | *J_SC_* [mA/cm^2^] | *FF* [%] | PCE [%] |
| --- | --- | --- | --- | --- |
| 1 | 1.15 | 25.30 | 82.82 | 24.13 |
| 2 | 1.14 | 25.17 | 80.98 | 23.17 |
| 3 | 1.13 | 24.75 | 82.04 | 23.02 |
| 4 | 1.14 | 24.87 | 81.12 | 22.90 |
| 5 | 1.14 | 25.20 | 81.36 | 23.42 |
| 6 | 1.14 | 25.16 | 80.88 | 23.13 |
| 7 | 1.16 | 25.35 | 82.21 | 24.07 |
| 8 | 1.14 | 25.22 | 82.03 | 23.62 |
| 9 | 1.14 | 25.16 | 82.24 | 23.49 |
| 10 | 1.13 | 25.30 | 80.96 | 23.09 |
| 11 | 1.14 | 25.24 | 80.36 | 23.05 |
| 12 | 1.15 | 25.26 | 81.29 | 23.63 |
| 13 | 1.15 | 25.09 | 80.43 | 23.11 |
| 14 | 1.14 | 25.07 | 80.37 | 23.04 |
| 15 | 1.14 | 25.02 | 81.74 | 23.39 |
| 16 | 1.13 | 24.70 | 81.52 | 22.78 |
| 17 | 1.15 | 25.13 | 81.93 | 23.73 |
| 18 | 1.13 | 25.32 | 80.85 | 23.17 |
| 19 | 1.14 | 24.85 | 82.70 | 23.39 |
| 20 | 1.15 | 25.07 | 82.47 | 23.83 |
| Average | 1.14 | 25.11 | 81.52 | 23.36 |

**Table S7.** The calculated *V_OC_* loss analysis results of the pristine and ISSP-treated PSCs.

| Sample | Eg [eV] | V_OC_^SQ^ [V] | ΔV_1_ [eV] | ΔV_2_ [V] | ΔV_3_ [V] | V_OC-Cal_ [V] | V_OC_ [V] |
| --- | --- | --- | --- | --- | --- | --- | --- |
| Pristine | 1.51 | 1.238 | 0.271 | 0.016 | 0.140 | 1.083 | 1.10 |
| ISSP | 1.51 | 1.237 | 0.270 | 0.006 | 0.064 | 1.166 | 1.16 |

**Table S8.** Fitted parameters of the TRPL curves of the corresponding perovskite films.

| Sample | A_1_ | τ_1_ [ns] | A_2_ | τ_2_ [ns] | τ_ave_ [ns] | R^2^ |
| --- | --- | --- | --- | --- | --- | --- |
| Pristine | 0.83 | 75.56 | 0.14 | 2693.51 | 99.96 | 0.97131 |
| ISSP-treated | 0.54 | 62.80 | 0.39 | 1585.06 | 218.40 | 0.98511 |

**Table S9.** The *J-V* parameters of the PSCs by dynamically spin-coated with different surface polishing agent.

| Sample | *V_OC_* [V] | *J_SC_* [mA/cm^2^] | FF [%] | PCE [%] |
| --- | --- | --- | --- | --- |
| Pristine | 1.10 | 24.63 | 77.06 | 20.88 |
| CB | 1.09 | 24.39 | 77.56 | 20.67 |
| TL | 1.10 | 24.39 | 78.10 | 21.01 |
| IPA | 1.11 | 24.56 | 80.59 | 22.02 |
| HFIP | 1.15 | 25.30 | 82.82 | 24.13 |

**Table S10.** Summary of the solvents' relative polarity to water.

| Solvent | Relative Polarity |
| --- | --- |
| Toluene (TL) | 0.099 |
| Chlorobenzene (CB) | 0.188 |
| Dimethylformamide (DMF) | 0.386 |
| Dimethylsulfoxide (DMSO) | 0.444 |
| 2-propanol (IPA) | 0.546 |
| 1,1,1,3,3,3-hexafluoropropan-2-ol (HFIP) | 0.969 |
| Water | 1 |

**Table S11.** The *J-V* parameters and the integrated *J_SC_* of Cs_0.05_(FA_0.83_MA_0.17_)_0.95_Pb(I_0.83_Br_0.17_)_3_ PSCs with and without ISSP treatment.

| Sample | *V_OC_* [V] | *J_SC_* [mA/cm^2^] | FF [%] | PCE [%] | Integrated *J_SC_* [mA/cm^2^] |
| --- | --- | --- | --- | --- | --- |
| Pristine | 1.10 | 21.97 | 77.36 | 18.69 | 20.97 |
| ISSP-treated | 1.16 | 21.82 | 80.81 | 20.53 | 21.07 |

**Table S12.** The *J-V* parameters and the integrated *J_SC_* of MAPbI_3_ PSCs with and without ISSP treatment.

| Sample | *V_OC_* [V] | *J_SC_* [mA/cm^2^] | FF [%] | PCE [%] | Integrated *J_SC_* [mA/cm^2^] |
| --- | --- | --- | --- | --- | --- |
| Pristine | 1.08 | 22.47 | 77.23 | 18.78 | 21.69 |
| ISSP-treated | 1.13 | 22.94 | 79.94 | 20.72 | 21.83 |

**Table S13.** The *J-V* parameters of FPSCs with and without ISSP treatment.

| Sample | *V_OC_* [V] | *J_SC_* [mA/cm^2^] | FF [%] | PCE [%] |
| --- | --- | --- | --- | --- |
| Pristine | 1.04 | 23.69 | 73.25 | 18.04 |
| ISSP-treated | 1.12 | 24.29 | 81.63 | 22.14 |

**References**

1. Y. Ge, F. Ye, M. Xiao, H. Wang, C. Wang, J. Liang, X. Hu, H. Guan, H. Cui, W. Ke, C. Tao, G. Fang, Internal Encapsulation for Lead Halide Perovskite Films for Efficient and Very Stable Solar Cells, Adv. Energy Mater. **2022**, 12, 2200361.

[2] S. Wu, J. Zhang, Z. Li, D. Liu, M. Qin, S. H. Cheung, X. Lu, D. Lei, S. K. So, Z. Zhu, A. K. Y. Jen, Modulation of Defects and Interfaces through Alkylammonium Interlayer for Efficient Inverted Perovskite Solar Cells, Joule **2020**, 4, 1248-1262.

[3] H. Sun, T. Liu, J. Yu, T.-K. Lau, G. Zhang, Y. Zhang, M. Su, Y. Tang, R. Ma, B. Liu, J. Liang, K. Feng, X. Lu, X. Guo, F. Gao, H. Yan, A monothiophene unit incorporating both fluoro and ester substitution enabling high-performance donor polymers for non-fullerene solar cells with 16.4% efficiency, Energy Environ. Sci. **2019**, 12, 3328-3337.

[4] X. Sun, Z. Li, X. Yu, X. Wu, C. Zhong, D. Liu, D. Lei, A. K. Y. Jen, Z. a. Li, Z. Zhu, Efficient Inverted Perovskite Solar Cells with Low Voltage Loss Achieved by a Pyridine-Based Dopant-Free Polymer Semiconductor, Angew. Chem. Int. Ed. **2021**, 60, 7227-7233.

[5] C. M. Sutter-Fella, D. W. Miller, Q. P. Ngo, E. T. Roe, F. M. Toma, I. D. Sharp, M. C. Lonergan, A. Javey, Band Tailing and Deep Defect States in CH_3_NH_3_Pb(I_1–x_Br_x_)_3_ Perovskites As Revealed by Sub-Bandgap Photocurrent, ACS Energy Lett. **2017**, 2, 709-715.

[6] W. Tress, M. Yavari, K. Domanski, P. Yadav, B. Niesen, J. P. C. Baena, A. Hagfeldt, M. Graetzel, Interpretation and evolution of open-circuit voltage, recombination, ideality factor and subgap defect states during reversible light-soaking and irreversible degradation of perovskite solar cells, Energy Environ. Sci. **2018**, 11, 151-165.

[7] J. Wang, J. Zhang, Y. Zhou, H. Liu, Q. Xue, X. Li, C.-C. Chueh, H.-L. Yip, Z. Zhu, A. K. Y. Jen, Highly efficient all-inorganic perovskite solar cells with suppressed non-radiative recombination by a Lewis base, Nat. Commun. **2020**, 11, 177.

[8] R. Su, Z. Xu, J. Wu, D. Luo, Q. Hu, W. Yang, X. Yang, R. Zhang, H. Yu, T. P. Russell, Q. Gong, W. Zhang, R. Zhu, Dielectric screening in perovskite photovoltaics, Nat. Commun. **2021**, 12, 2479.

[9] B. Chen, H. Chen, Y. Hou, J. Xu, S. Teale, K. Bertens, H. Chen, A. Proppe, Q. Zhou, D. Yu, K. Xu, M. Vafaie, Y. Liu, Y. Dong, E. H. Jung, C. Zheng, T. Zhu, Z. Ning, E. H. Sargent, Passivation of the Buried Interface via Preferential Crystallization of 2D Perovskite on Metal Oxide Transport Layers, Adv. Mater. **2021**, 33, 2103394.

[10] W. Tress, N. Marinova, O. Inganas, M. K. Nazeeruddin, S. M. Zakeeruddin, M. Graetzel, Predicting the Open-Circuit Voltage of CH_3_NH_3_PbI_3_ Perovskite Solar Cells Using Electroluminescence and Photovoltaic Quantum Efficiency Spectra: the Role of Radiative and Non-Radiative Recombination, Adv. Energy Mater. **2015**, 5, 1400812.

[11] K. Vandewal, K. Tvingstedt, A. Gadisa, O. Inganas, J. V. Manca, Relating the open-circuit voltage to interface molecular properties of donor:acceptor bulk heterojunction solar cells, Phys. Rev. B **2010**, 81, 125204.

[12] H. Sun, S. Wang, S. Qi, P. Wang, R. Li, B. Shi, Q. Zhang, Q. Huang, S. Xu, Y. Zhao, X. Zhang, Surface Defects Management by In Situ Etching with Methanol for Efficient Inverted Inorganic Perovskite Solar Cells, Adv. Funct. Mater. **2023**, 33, 2213913.

[13] S. Fu, J. Le, X. Guo, N. Sun, W. Zhang, W. Song, J. Fang, Polishing the Lead-Poor Surface for Efficient Inverted CsPbI_3_ Perovskite Solar Cells, Adv. Mater. **2022**, 34, 2205066.

[14] H. Wang, L. Deng, Y. Pan, X. Zhang, X. Li, Y. Wang, Y. Wang, Y. Liu, X. Yue, Z. Shi, C. Li, K. Liu, T. Hu, Z. Liang, C. Tian, J. Wang, A. Yu, X. Zhang, Y. Yang, Y. Zhan, Green Solvent Polishing Enables Highly Efficient Quasi-2D Perovskite Solar Cells, ACS Appl. Mater. Inter. **2023**, 15, 36447-36456.

[15] J. Wang, K. Wang, C. Zhang, S. Liu, X. Guan, C. Liang, C.-C. Chen, F. Xie, Surface Cleaning and Passivation Strategy for Durable Inverted Formamidinium-Cesium Triiodide Perovskite Solar Cells, Adv. Energy Mater. **2023**, 13, 2302169.

[16] W. Kong, C. Zhao, J. Xing, Y. Zou, T. Huang, F. Li, J. Yang, W. Yu, C. Guo, Enhancing Perovskite Solar Cell Performance through Femtosecond Laser Polishing, Sol. RRl **2020**, 4, 2000189.

[17] S. Chen, Y. Liu, X. Xiao, Z. Yu, Y. Deng, X. Dai, Z. Ni, J. Huang, Identifying the Soft Nature of Defective Perovskite Surface Layer and Its Removal Using a Facile Mechanical Approach, Joule **2020**, 4, 2661-2674.

[18] M. Yuan, H. Ma, Q. Dong, X. Wang, L. Zhang, Y. Yin, Z. Ying, J. Guo, W. Shang, J. Zhang, Y. Shi, Chemical polishing and sub-surface passivation of perovskite film towards high efficiency solar cells, Nano Energy **2024**, 121, 109192.

[19] L. Zhao, Q. Li, C.-H. Hou, S. Li, X. Yang, J. Wu, S. Zhang, Q. Hu, Y. Wang, Y. Zhang, Y. Jiang, S. Jia, J.-J. Shyue, T. P. Russell, Q. Gong, X. Hu, R. Zhu, Chemical Polishing of Perovskite Surface Enhances Photovoltaic Performances, J. Am. Chem. Soc. **2022**, 144, 1700-1708.

[20] M. Kedia, M. Rai, H. Phirke, C. A. Aranda, C. Das, V. Chirvony, S. Boehringer, M. Kot, M. M. Byranvand, J. I. Flege, A. Redinger, M. Saliba, Light Makes Right: Laser Polishing for Surface Modification of Perovskite Solar Cells, ACS Energy Lett. **2023**, 8, 2603-2610.

[21] H. Back, G. Kim, H. Kim, C.-Y. Nam, J. Kim, Y. R. Kim, T. Kim, B. Park, J. R. Durrant, K. Lee, Highly stable inverted methylammonium lead tri-iodide perovskite solar cells achieved by surface re-crystallization, Energy Environ. Sci. **2020**, 13, 840-847.

[22] X. Xiao, C. Bao, Y. Fang, J. Dai, B. R. Ecker, C. Wang, Y. Lin, S. Tang, Y. Liu, Y. Deng, X. Zheng, Y. Gao, X. C. Zeng, J. Huang, Argon Plasma Treatment to Tune Perovskite Surface Composition for High Efficiency Solar Cells and Fast Photodetectors, Adv. Mater. **2018**, 30, 1705176.
